# Sociodemographic and dietary predictors of maternal and placental mycoestrogen concentrations in a US pregnancy cohort

**DOI:** 10.1038/s41370-024-00722-6

**Published:** 2024-10-04

**Authors:** Carolyn W. Kinkade, Anita Brinker, Brian Buckley, Olivia Waysack, I. Diana Fernandez, Amber Kautz, Ying Meng, Huishan Shi, Jessica Brunner, Pamela Ohman-Strickland, Susan W. Groth, Thomas G. O’Connor, Lauren M. Aleksunes, Emily S. Barrett, Zorimar Rivera-Núñez

**Affiliations:** 1 Environmental and Occupational Health Sciences Institute, Rutgers University, Piscataway, NJ, USA.; 2 Department of Public Health Sciences, University of Rochester Medical Center, Rochester, NY, USA.; 3 School of Nursing, University of Rochester, Rochester, NY, USA.; 4 Department of Obstetrics and Gynecology, University of Rochester Medical Center, Rochester, NY, USA.; 5 Department of Biostatistics and Epidemiology, Rutgers School of Public Health, Piscataway, NJ, USA.; 6 Department of Psychiatry, University of Rochester, Rochester, NY, USA.; 7 Ernest Mario School of Pharmacy, Rutgers University, Piscataway, NJ, USA.; 8 These authors contributed equally: Emily S. Barrett, Zorimar Rivera-Núñez.

**Keywords:** Mycotoxin, Zearalenone, Mycoestrogen, Zeranol, Endocrine disruption, Pregnancy

## Abstract

**BACKGROUND:**

Zearalenone (ZEN) is a mycotoxin contaminating grains and processed foods. ZEN alters nuclear estrogen receptor α/β signaling earning its designation as a mycoestrogen. Experimental evidence demonstrates that mycoestrogen exposure during pregnancy is associated with altered maternal sex steroid hormones, changes in placental size, and decreases in fetal weight and length. While mycoestrogens have been detected in human biospecimens worldwide, exposure assessment of ZEN in US populations, particularly during pregnancy, is lacking.

**OBJECTIVE:**

To characterize urinary and placental concentrations of ZEN and its metabolites in healthy US pregnant people and examine demographic, perinatal, and dietary predictors of exposure.

**METHODS:**

Urine samples were collected in each trimester from pregnant participants in the UPSIDE study and placenta samples were collected at delivery (Rochester, NY, *n* = 317). We used high performance liquid chromatography and high-resolution tandem mass spectrometry to measure total urinary (ng/ml) and placental mycoestrogens (ng/g). Using linear regression and linear mixed effect models, we examined associations between mycoestrogen concentrations and demographic, perinatal, and dietary factors (Healthy Eating Index [HEI], ultra-processed food [UPF] consumption).

**RESULTS:**

Mycoestrogens were detected in 97% of urines (median 0.323 ng/ml) and 84% of placentas (median 0.012 ng/g). Stability of urinary mycoestrogens across pregnancy was low (ICC: 0.16–0.22) and did not correlate with placental levels. In adjusted models, parity (multiparous) and pre-pregnancy BMI (higher) predicted higher urinary concentrations. Birth season (fall) corresponded with higher placental mycoestrogens. Dietary analyses indicated that higher HEI (healthier diets) predicted lower exposure (e.g., Σmycoestrogens %Δ −2.03; 95%CI −3.23, −0.81) and higher percent calories from UPF predicted higher exposure (e.g., Σmycoestrogens %Δ 1.26; 95%CI 0.29, 2.24).

**IMPACT:**

• The mycotoxin, zearalenone (ZEN), has been linked to adverse health and reproductive impacts in animal models and livestock. Despite evidence of widespread human exposure, relatively little is known about predictors of exposure. In a pregnant population, we observed that maternal ZEN concentrations varied by maternal pre-pregnancy BMI and parity. Consumption of ultra-processed foods, added sugars, and refined grains were linked to higher ZEN concentrations while healthier diets were associated with lower levels. Our research suggests disparities in exposure that are likely due to diet. Further research is needed to understand the impacts of ZEN on maternal and offspring health.

## INTRODUCTION

Zearalenone (ZEN) is one of the most common mycotoxin contaminants in global food supplies [1]. ZEN, a secondary metabolite of *Fusarium* fungi, contaminates cereal grains (e.g., maize, wheat, barley, oats, sorghum), and is widely detected in processed foods (e.g., pasta, cereal, bread) [1–3]. ZEN is metabolized by 3α- and 3β-hydroxysteroid dehydrogenases into primary metabolites alpha-zearalenol (α-ZOL) and beta-zearalenol (β-ZOL) in animals and humans [4]. In the United States, zeranol (ZER), a synthetic derivative of ZEN, is administered to livestock as a growth promoter, making meat another potential source of human exposure [4]. Human exposure studies have detected ZEN and its metabolites in populations worldwide, with levels varying by biospecimen type (plasma, serum, breast milk, urine) and geographical region [5–9]. Although very little epidemiological research has examined ZEN in relation to human health outcomes, a large literature in experimental models and livestock clearly demonstrates its carcinogenic, genotoxic, immunotoxic, and endocrine disrupting effects [4, 10]. Studies in animal models (including pigs, sheep, cows, rats, mice, and hens) show that after ingestion, ZEN and metabolites are distributed to the liver as well as reproductive organs and the placenta [11–14]. The biological half-life is estimated between 3–86 h depending on species and route of administration [3].

Notably, the chemical structures of ZEN, ZER, and their metabolites closely resemble 17β estradiol (E_2_) allowing them to directly bind to nuclear estrogen receptors α (ER-α) and β (ER-β), resulting in their designation as “mycoestrogens”. In fact, mycoestrogens have greater estrogenic potency than other endocrine disruptors including genistein, bisphenol A, and phthalates [15–17]. In mice, rats, and pigs, exposure to mycoestrogens altered uterine ER-α and ER-β receptor density, hormonal signaling, reproductive organ weight, and fertility [18–23]. Impacts extend into pregnancy with animal studies demonstrating that mycoestrogen exposure can lead to adverse perinatal outcomes including alterations in sex steroid hormones and placental development, reduced offspring weight and length, skeletal malformations, and even fetal death [24–28]. Few studies have examined exposure in humans during pregnancy [29, 30], though it was recently reported that mycoestrogens are associated with altered serum sex steroid hormone concentrations during pregnancy [31].

Given the prevalence of ZEN in the food supply and its demonstrated toxicity in animal models, 16 countries have enacted regulations to limit human consumption [3]. Based on its endocrine disrupting properties, ZER is banned in the European Union (EU) while the tolerable daily intake for ZEN is 0.25 μg/kg/b wt [4, 32]. In the US, ZER is in widespread use with an acceptable daily intake of 1.25 μg/kg/day [33] and there is no regulatory limit for ZEN. Making concerns about human exposure more urgent, mycoestrogen levels are expected to rise with climate change. ZEN is most often detected in countries with warm wet climates [34]. The proliferation of *Fusarium* and subsequent production of mycotoxins (including ZEN) is influenced by rainfall, humidity, temperature, and carbon dioxide [35]. Moreover, the geographic locations of mycotoxin production are predicted to shift due to climate change [36]. Fungal growth and mycotoxin production, and thus food contamination and exposure are inextricably linked to climate [35, 37] and the United Nations Environmental Program has identified mycotoxin contamination of the food supply as a research and mitigation priority [38].

Mycoestrogen exposure is an emerging threat to public health and this exposure has not been well characterized in pregnancy. In perinatal epidemiology, maternal exposure is often interpreted to represent fetal exposure, though this is not proven for each chemical [39]. We know that these chemicals do impact pregnant individuals and we seek to identify potentially vulnerable groups and ways to reduce exposure [31] Therefore, to fill the knowledge gap regarding factors influencing developmental exposure to mycoestrogens in humans, the objectives of the present study were to:(1) characterize maternal and fetal exposure to mycoestrogens through serial exposure assessment, (2) determine the relationship between maternal urine and placental concentrations, (3) identify predictors of exposure (e.g., sociodemographic, dietary) that may inform future intervention strategies.

## METHODS

### Study sample

The current analysis uses data from pregnant participants in the Understanding Pregnancy Signals and Infant Development (UPSIDE) cohort study (Rochester, N.Y., USA) [40]. Between 2015 and 2019, the University of Rochester Medical Center and associated clinics recruited 326 pregnant participants based on the following criteria:(1) ≥18 years of age, (2) in the first trimester of pregnancy, (3) singleton pregnancy, (4) no major substance use issues or history of psychosis, (5) no major endocrine disorder (such as polycystic ovary syndrome), and (6) able to communicate in English. Study participants provided signed consent, and Institutional Review Boards at the University of Rochester and Rutgers University approved all study activities. Participants completed study visits in each trimester consisting of biospecimen collection and questionnaires on demographics, lifestyle, and health history. Placentas were collected at delivery. Of the 326 participants who enrolled in the UPSIDE study, 317 provided at least one urine specimen. Specifically, 301, 283, and 281 participants provided urine in the 1st, 2nd, and 3rd trimesters, respectively (Supplementary Fig. 1). One 1st trimester measurement that did not pass laboratory quality control standards was excluded, leaving 258 participants with mycoestrogen concentrations in all trimesters. Additionally, 274 placentas were collected at delivery, three were excluded from further study due to placenta abnormalities. The number of participants that contributed data to our primary analysis of urinary or placental predictors was 292.

### Mycoestrogen concentrations

At three study visits across pregnancy (12.2 ± 1.3, 21.2 ± 1.8, 31.4 ± 1.9 weeks gestation) urine samples were collected. Specific gravity was measured using a refractometer (Atago 4410 PAL-10S Digital Hand-Held Pocket Urine Specific Gravity Refractometer, Tokyo, Japan), after which urine samples were frozen at −80 °C. Trained study coordinators collected placentas following standard protocols previously described [41]. Within 1 h of birth, study coordinators took samples of the fresh core villous tissue while leaving the maternal decidua surface intact. ~30 g of tissue was frozen in liquid nitrogen and stored at −80 °C. Detailed methods for placental processing have been previously described [42]. Urine and placenta samples were transferred on dry ice to the Environmental and Occupational Health Sciences Institute at Rutgers University for analysis.

Urinary (total; unconjugated plus conjugated) and placental (total) mycoestrogen concentrations (6 analytes: α-ZOL, β-ZOL, α-zearalanol (α-ZAL/ZER), β-zearalanol (β-ZAL), zearalanone (ZAN), and ZEN) were analyzed by ultra high-performance liquid chromatography-tandem mass spectrometry (UPLC-MS/MS) using previously published methods [42, 43]. In short, urine (0.4 ml) or placenta (~200 mg, homogenized) was combined with internal standards (D_6_-ZEN and D_7_- α-ZOL [Toronto Research Chemicals North York, Ontario, Canada]), sodium acetate buffer (pH 4.6 [Honeywell Fluka, Morris Plains, NJ, USA]), and deconjugation enzymes (β-glucuronidase, type HP-2, with sulfatase [Sigma-Aldrich #G7017, St. Louis, MO, USA]) for overnight incubation, in parallel with matrix matched calibration curves. Mycoestrogens were extracted with two sequential solid phase extraction steps (1st (urine): Chem ElutTM cartridges (1 ml, [Agilent Santa Clara, CA, USA], eluted with 3 × 2 ml methyl tert-butyl ether [MTBE]) or 1st (placenta): Chem ElutTM cartridges (3 ml, [Agilent Santa Clara, CA, USA], eluted with 3 × 5 ml MTBE; 2nd: Discovery DSC-NH2 cartridges [Sigma-Aldrich] with methanol) with intermittent drying under a nitrogen stream. Mycoestrogens were redissolved in HPLC solvent (for urine: 200 μl, for placenta 50 μl; 50% water, 25% acetonitrile, 25% methanol) and transferred to HPLC vials. Calibration curve samples were prepared from stock solutions (0.025–5.0 ng/ml) dissolved in acetonitrile (Sigma Aldrich ZEN: #Z2125, α-ZOL: #Z0166, β-ZOL: #Z2000, α-ZAL: #Z0292, ZAN: #Z0167, β-ZAL: #Z0417). Each run included blanks, quality control standards (analytes and the internal standard at 0.1 ng/ml in acetonitrile), and calibrator samples (0.025 ng/ml in matrix, duplicates). Concentrations were assessed by UPLC-MS/MS using a Dionex UltiMate 3000 UHPLC interfaced to a Thermo Scientific Q Exactive HF Hybrid Quadrupole-Orbitrap by Thermo Fisher Scientific (Waltham, MA, USA), run time parameters were previously published and details on quantitation are provided in the supplementary materials [42]. For analytes detected in >75% of samples, samples below the limit of detection were assigned a value of LOD/√(2) [44].

To standardize for urine dilution, we used the Boeniger formula: Pc = P[(SGm_tri_−1)/(SG−1)] where Pc is the specific gravity corrected analyte concentration, P is the mycoestrogen analyte concentration [45]. SGm_tri_ is the trimester median specific gravity for the UPSIDE cohort, and SG is the individual’s urine specific gravity for the sample.

### Estimating unconjugated and conjugated mycoestrogens

In phase 2 metabolism, mycoestrogens are conjugated by UDP-glucuronosyl transferases and sulfotransferases to produce glucuronide and sulfate conjugates presumably rendering them unable to interfere with ER α/β activity [3]. Therefore to understand endocrine disrupting potential of this exposure, we estimated the conjugated fraction in a subset of samples (*n* = 30, 10 per trimester). The subset was chosen because these participants had detectable levels of each analyte. Samples were prepared according to methods described above, except we omitted the enzymatic digestion step when measuring unconjugated mycoestrogens. Samples were prepared and quantified in parallel to assess percent unconjugated mycoestrogens relative to the total (unconjugated plus conjugated) concentrations.

### Sociodemographic and perinatal predictors of mycoestrogen concentrations

Sociodemographic and perinatal variables were collected from questionnaires completed at prenatal visits. These included maternal age (categorized here as <25, 25–29, 30–34, ≥35 years), race/ethnicity (Non-Hispanic White, Non-Hispanic Black, Other race [Asian, Pacific Islander, Mixed Race, and Other], Hispanic), education (less than high school/high school, some college/bachelors, and any post-graduate education), parity (nulliparous/parous), use of social services during pregnancy (any vs. no use of Medicaid, the Special Supplemental Nutrition Program for Women, Infants, and Children (WIC); or other public assistance), marital status (categorized here as married/living as married vs single/divorced/widowed/separated). We examined race/ethnicity as a proxy for structural racism which may affect access to resources including food [46]. Maternal height and weight at earliest prenatal appointment were used to calculate early pregnancy BMI (kg/m^2^) as a proxy for pre-pregnancy weight [47]. Because fungal growth on crops depends on weather and climate, mycoestrogen food contamination may follow seasonal patterns [37]. Season of biospecimen collection (urine or placenta) was categorized as spring (March, April, May), summer (June, July, August); fall (September, October, November), and winter (December, January, February). Finally, fetal sex differences in placental levels of environmental pollutants have been detected [48] and fetal-sex-specific impacts on health endpoints are often reported, therefore we considered fetal sex which was collected from the medical record.

### Dietary predictors

As mycoestrogen exposure is considered to occur almost exclusively through contaminated foods in the general population (non-occupational exposure), we evaluated potential dietary predictors of exposure. A trained nutritionist with knowledge of regional foods, preparation techniques, and food in the marketplace, conducted 1–3 24-h dietary recalls per participant in mid-late pregnancy over the telephone. The United States Department of Agriculture (USDA)’s Automated Multiple Pass Method was used [49] to obtain diet consumed within prior 24 h. The recalls were then entered into the Nutrition Data System for Research software (NDSR, 2017 version, University of Minnesota Nutrition Coordinating Center, Minneapolis, MN) to derive nutrient intake [50]. Here we focus on participants with two recalls during the 2nd trimester (*n* = 172) [49]. For participants with two recalls, the second occurred 3–30 days after the first. Based on these recalls, we calculated (1) overall energy intake (kcal/day), (2) Healthy Eating Index (HEI), and (3) ultra-processed food (UPF) consumption, (4) percent of protein from animal sources, (5) percent of protein from vegetable sources.

#### Overall energy intake.

The National Cancer Institute (NCI) has developed a statistical method to model aspects of usual dietary intake when the dietary assessment method is a Food Frequency Questionnaire (FFQ) or one to two 24-h dietary recalls [51]. This method was used to generate estimates of micro and macronutrient intake from food. In this analysis energy intake is estimated daily energy (kcals/day) from diet only.

#### The healthy eating index (HEI).

The HEI is a measure of diet quality used to assess how well a set of foods consumed aligns with key recommendations and dietary patterns published in the *Dietary Guidelines for Americans* (*DGA*) [52]. The HEI-2015 was used to score dietary recalls as the closest timing to our data collection (2015–2019). The maximum score is 100 and is derived from nine adequacy and four moderation food components; a higher score indicates closer adherence to the DGA and a healthier diet. The HEI-2015 adequacy components include [maximum points] total fruit [5], whole fruit [5], total vegetable [5], greens and beans [5], whole grains [10], dairy [10], total protein [5], seafood and plant protein [5], fatty acids [10] and the moderation components include refined grains [10], sodium [10], added sugar [10], and saturated fats [10]. The scoring standards for adequacy sub-scores are calculated based on volume of food group per 1000 kcal consumed. HEI-moderation sub-scores are calculated based on % of energy (added sugars or saturated fats) or volume/weight per 1000 kcal (refined grains, sodium). HEI total and sub-scores (adequacy and moderation) were calculated for each dietary recall. The HEI scores from two recalls from the second trimester were averaged.

#### Ultra-processed foods (UPF).

UPF are highly processed foods that contain ingredients intended to extend shelf life and make the food hyper palatable [53]. Our main measure of UPF was percent of calories in the diet deriving from UPF (UPF%). To calculate UPF%, first, unique food lists were compiled from participants’ dietary recalls and independently coded according the NOVA guidelines by two members of the research team [53]. The NOVA guidelines (not an acronym) are a system of classifying foods by the extent, nature, and purpose of food processing [53]. Differences in coding were resolved by a third rater. For composite foods (e.g., tacos, sandwiches), we disaggregated the components which were then individually coded. Based on those classifications, we determined the total calories coming from UPF. Finally, daily UPF% was calculated as (UPF calories/raw total calories)*100. The UPF% from two recalls from the second trimester was averaged.

#### Protein source.

Given that prior literature links mycoestrogen exposure to meat consumption we examined protein source [5, 54]. Grams of total protein, animal protein, and vegetable protein were abstracted from NDSR output. Percentage of protein from animal or vegetable source was calculated for each participant dietary recall. Protein source variables were averaged from two dietary recalls for each participant.

### Statistical analysis

Based on the prior literature, we created a composite measure of total mycoestrogen exposure (Σmycoestrogens) by summing analyte concentrations (i.e., ZEN, aZOL, bZOL, aZAL, bZAL, ZEN) from an individual’s sample [5]. We examined descriptive statistics (including percent above LOD, median, geometric mean, geometric standard deviation, and percentiles) for all analytes. Specific-gravity adjusted concentrations were log-transformed to improve normality. To examine reliability of mycoestrogen concentrations, we calculated intraclass correlation (ICC; two-way, consistency) across time periods (1st/2nd, 2nd/3rd, 1st/3rd, 1st, 2nd, 3rd). We used chi-square tests to examine independence of the categorical predictor variables.

In our primary analysis, we fitted linear mixed effect models with log-transformed urinary concentrations as the response variable and sociodemographic and perinatal factors as predictors (fixed effects). We considered three outcomes (concentrations of aZOL, ZEN, and Σmycoestrogens) as the rest of the analytes were detectable in only a small fraction of samples. We first created individual models for each predictor including a random effect for each participant. Additionally, we examined mutually adjusted models that included all predictors that were significant in unadjusted models (urine or placenta models) at *p* < 0.05. All of the following covariates are included in the mutually adjusted models for urine were: maternal age, BMI, ethnicity/race, education, parity, use of social services, marital status, fetal sex, season of urine collection, and sampling time (trimester: 1st, 2nd, 3rd). We additionally considered models with sampling time as a continuous variable (gestational weeks) and results were similar. In preliminary analysis, we additionally considered alcohol use and smoking in pregnancy, but they were dropped from further consideration due to lack of association with any concentrations. Due to the high variability of concentrations across pregnancy, our primary analysis consisted of participants who contributed urine samples in all trimesters; secondarily we analyzed predictors amongst participants who had at least one urine sample (*n* = 317).

We additionally considered predictors of placental mycoestrogens using generalized linear models or logistic regression models. ZEN (as a dichotomous variable (detect/non-detect) and Σmycoestrogens (continuous) were the response variables in these models, because individual placental metabolites were detected in <75% of samples. In the placental models where the response variables were log-transformed Σmycoestrogens concentrations, we used generalize linear models. We additionally used logistic regression models with ZEN as a binary variable (non-detect/detect). Predictors were first considered individually in unadjusted models. Subsequently, we fitted mutually adjusted models including maternal age, BMI, ethnicity/race, education, parity, use of social services, marital status, fetal sex, and season of placenta collection. Covariates were included if they were associated with mycoestrogen concentrations (urinary or placental) at *p* < 0.05. We examined independence of covariates with chisquare test.

For both urine and placental predictors, for ease of interpretation, we calculated a ratio (concentration compared to reference group) by exponentiating the beta estimate, and confidence intervals limits. Ratios <1.0 and >1.0 indicate lower or higher exposure than the reference group, respectively [55, 56]. We additionally report ß estimates and 95% confidence intervals in supplementary material. We performed multiple sensitivity analysis. First, we examined predictors of aZOL concentrations in participants who had detectable aZOL (*n* = 252 participants, *n* = 649 measurements), to eliminate any potential bias caused by replacing missing values with LOD√2. Second, we performed a sensitivity analysis including three placentas that were partially delivered and which were excluded from the primary analysis.

In the analysis of dietary predictors, we examined associations between dietary factors (total daily energy intake, HEI total, HEI sub-scores [total fruit, whole fruit, total vegetable, greens and beans, whole grains, dairy, total protein, seafood and plant protein, fatty acids, refined grains, sodium, added sugar, saturated fats], UPF%, % of protein from animal sources, % of protein from vegetable sources) and 2nd trimester mycoestrogen concentrations to match timing of exposure measurement with duplicate dietary recalls. We examined Pearson correlations between all dietary parameters considered. Dietary factors as predictors were considered individually in unadjusted linear models. In adjusted models, we include covariates identified in predictors analysis (maternal age, race/ethnicity, parity, education, season of urine collection, marital status, use of social services). For ease of interpretation of regression results, HEI-moderation sub-scores estimates (sodium, added sugars, refined grains, saturated fats) were multiplied by −1 so that higher score indicated higher consumption of that dietary component. In a sensitivity analysis of dietary predictors, to account for correlation of HEI subscores, we considered models where we additionally adjusted for total HEI score minus the subscore predictor [57, 58]. In a post hoc analysis, we examined differences in diet quality by levels of sociodemographic predictor of urinary mycoestrogen concentrations.

All analyses were performed in R Studio (Version 2023.06.1 + 524)

## RESULTS

### Descriptive and bivariate associations

The analytic sample included 292 participants. Of these, 258 completed all 3 prenatal study visits, contributing 774 urine specimens; 271 participants contributed placenta at delivery (Supplementary Fig. 1), and 237 contributed samples at all 4 time points. Most participants enrolled in the study were between 25 and 34 years of age (70.6%), non-Hispanic White (57.5%), married or living as married (58.6%), multiparous (66.1), and had some college education or greater (61.7%) (Table 1). Over half of participants had BMI indicative of overweight (26.4%) or obesity (30.1%) at the beginning of pregnancy and 55% reported use of social services (WIC, Medicaid, public assistance) during pregnancy. Less than 10% of births (*n* = 15) were preterm. The relationship between predictor variables is reported in Supplementary Table. 1. Notably, there were significant association between variables, for example race and ethnicity was related to pre-pregnancy BMI, marital status, use of social services, and preterm birth, Participants included in the dietary predictors analysis (*n* = 172), reported average energy intake of 2161.6 ± 321.3 kcal/day, with 53.8% of calories coming from UPF. Average HEI scores were 54.9 ± 13.8. More dietary protein was derived from animal sources (61.6%) than vegetable sources (38.4%).

**Fig. 1:**
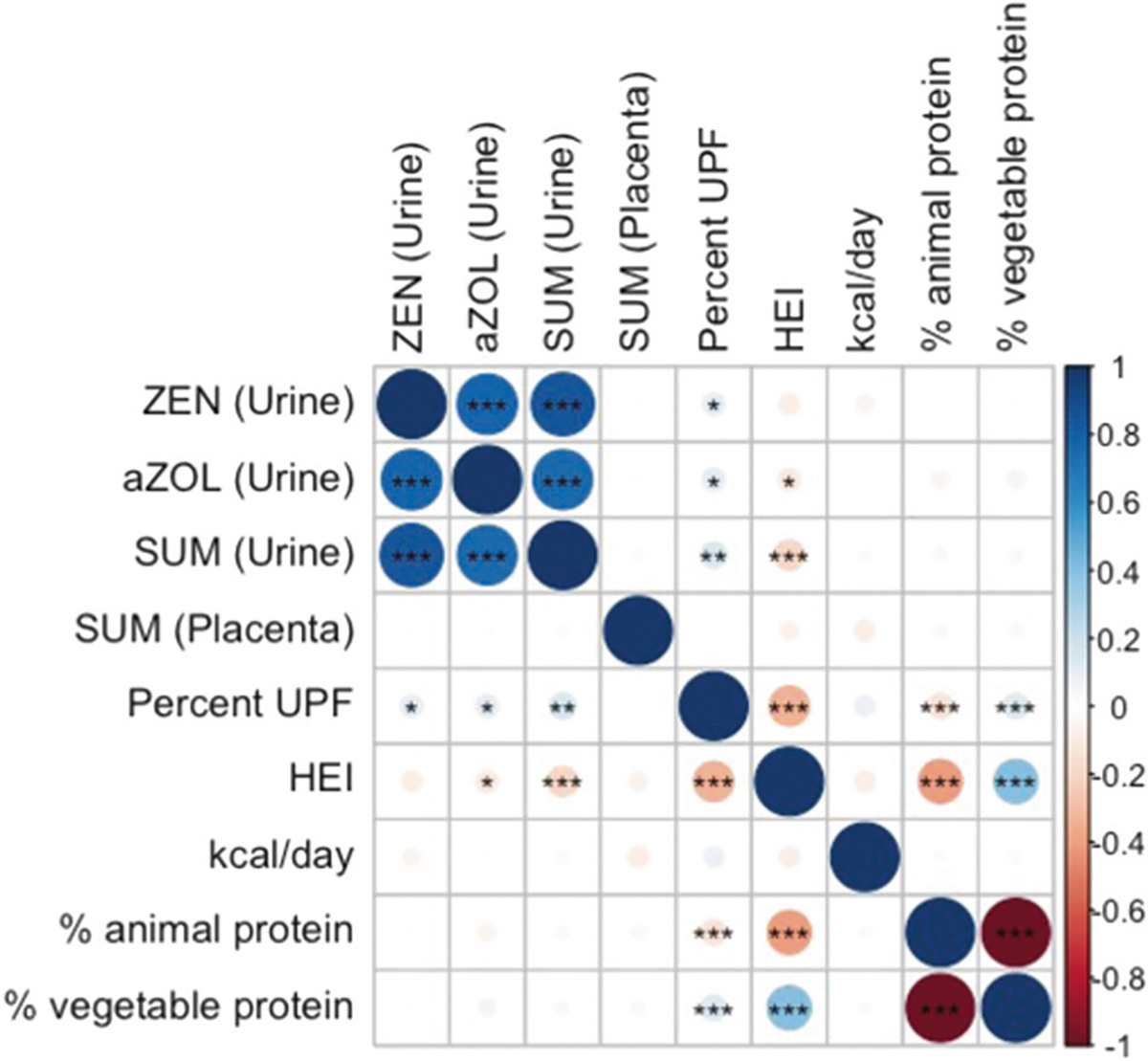
Spearman correlation between log-transformed mycoestrogen concentration in urine (ng/ml) and placenta (ng/g) and dietary parameters in the UPSIDE cohort. ^†^The N number for urine measurements is 258 (3 measurements per person), placental measurement is 271, dietary measurements is 172. Significance is indicated by *(*p* < 0.05), **(*p* < 0.01), ***(*p* < 0.001). aZOL alphazearalenol, kcal kilocalories, HEI healthy eating index, UPF ultraprocessed foods, SUM sum of mycoestrogen analytes, ZEN zearalenone.

ZEN was detected in 95.6% of urine samples (Table 2). Based on the sub-analysis of 30 participants, an estimated 82.5% of ZEN in urine was conjugated (Supplementary Fig. 2). The primary metabolite in humans, α-ZOL, was detected in 83.9% of urine samples and median α-ZOL concentrations tended to be higher than ZEN concentrations. Metabolites β-ZAL, β-ZOL, α-ZAL (ZER), and ZAN were detected in less than 50% of urine samples (Table 2, Supplementary Table 2). Concentrations of ZEN were highly correlated with concentrations of α-ZOL and Σmycoestrogens (*r* = 0.79–0.89; Fig. 1 and Supplementary Table 3). In urine samples, median mycoestrogen concentrations increased across pregnancy (e.g., ZEN, 1st: 0.094, 2nd: 0.101, 3rd: 0.160 ng/ml), however the ICCs across three timepoints were low (α-ZOL: 0.22, ZEN: 0.20; Σmycoestrogens: 0.16). Consistency of measurements was higher when comparing two contiguous time points (e.g., ZEN 1st and 2nd ICC: 0.26 95% CI: 0.14, 0.37) and lower comparing 1st to 3rd trimester (e.g., ZEN ICC 0.03 95% CI: 0.00, 0.23) (data not shown). In each trimester, spearman correlation between α-ZOL and the parent compound (ZEN) was high (e.g., 2nd ZEN/α-ZOL *r* = 0.79 *p* < 0.001) (Supplementary Fig. 3).

ZEN was detected in 59.4% of placental samples and β-ZAL, β-ZOL, α-ZAL, ZAN were below LOD in at least 70% of samples (Table 2). Σmycoestrogens (median 0.012 ng/g) were found in 84.1% of specimens. Placenta concentrations did not correlate with urinary concentrations, though correlations between matrixes were slightly higher when comparing 3rd trimester urine to placental concentrations (Σmycoestrogens 1st/Σmycoestrogens placenta *r* = 0.00; α-ZOL 3rd/ Σmycoestrogens placenta *r* = 0.08; Supplementary Fig. 3).

### Models examining predictors of urinary mycoestrogen exposure

#### Unadjusted models.

**Fig. 2:**
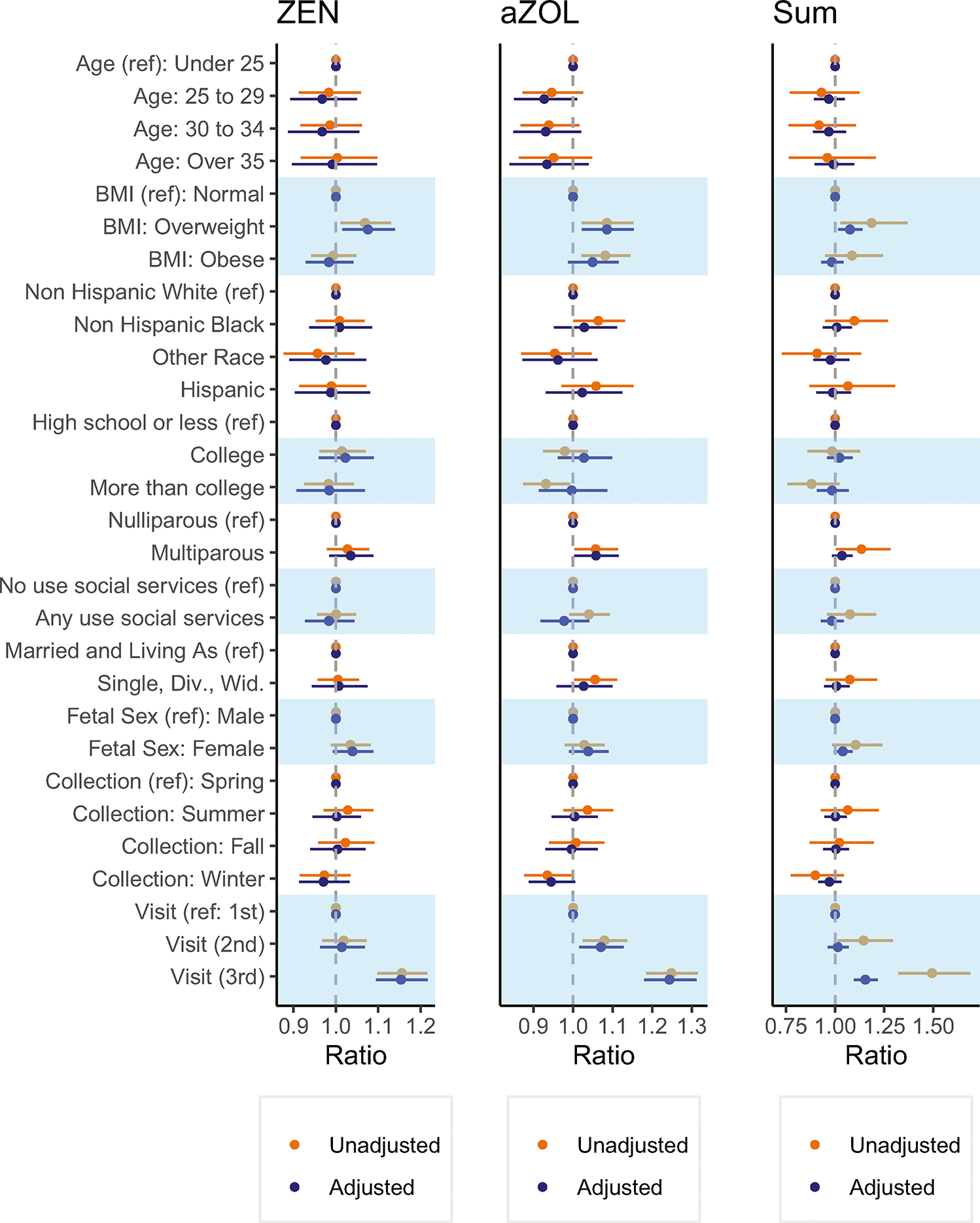
Ratio of urinary mycoestrogen concentrations (ng/ml) by sociodemographic, lifestyle, and perinatal characteristics of UPSIDE participants (*N* = 258). ^†^Ratios were calculated as the exponent of the beta coefficient. All mycoestrogen concentrations are specific gravity adjusted and missing values were assigned LOD/√2. The mutually adjusted models (*n* = 254) were adjusted for all considered predictors (i.e., maternal age, early pregnancy BMI, race/ethnicity, parity, use of social services, marital status, fetal sex, season of urine collection), and gestational weeks at urine collection. aZOL alpha-zearalenol, ZEN zearalenone, SUM sum of mycoestrogen analytes.

Ratios and beta estimates are reported in Fig. 2 and Supplementary Table 4. In analyses determining sociodemographic and perinatal predictors of exposure, having a BMI indicative of being overweight at the start of pregnancy was associated with higher exposure (Σmycoestrogens ratio 1.19; 95% CI: 1.03, 1.37, relative to underweight/normal BMI). Multiparous participants had higher α-ZOL concentrations than primiparous participants (ratio 1.06 95%CI: 1.00, 1.11). Samples collected at study visits later in pregnancy had higher concentrations compared to earlier visits (3rd trimester ZEN ratio 1.16; 95%CI: 1.10, 1.22).

#### Adjusted models.

In adjusted models, having a BMI indicating overweight at the start of pregnancy (ZEN ratio 1.08; 95%CI 1.01, 1.14, α-ZOL ratio 1.09 95%CI 1.02, 1.15), multiparity (ZEN ratio 1.03 95%CI 0.98, 1.09; α-ZOL ratio 1.06; 95%CI 1.00, 1.12), and sampling time (3rd trimester ZEN ratio 1.15; 95%CI: 1.09, 1.22) were significant predictors (Fig. 2, Supplementary Table 5). Maternal age, race/ethnicity, education, use of social support, fetal sex, and marital status were not significant predictors. In sensitivity analyses that did not include imputation of values below the LOD, results were virtually unchanged (Supplementary Table 6).

We conducted secondary analysis considering predictors amongst participants with at least one urine specimen (*n* = 317) rather than a complete set of samples. Unadjusted ratios and beta estimates are reported in Supplementary Table 7. In this sample, unadjusted associations between mycoestrogens and education, marital status, and race and ethnicity, were not significant in mutually adjusted models (Supplementary Table 8). Additionally, samples collected later in pregnancy had higher concentrations.

### Models examining predictors of placental mycoestrogen exposure

**Fig. 3:**
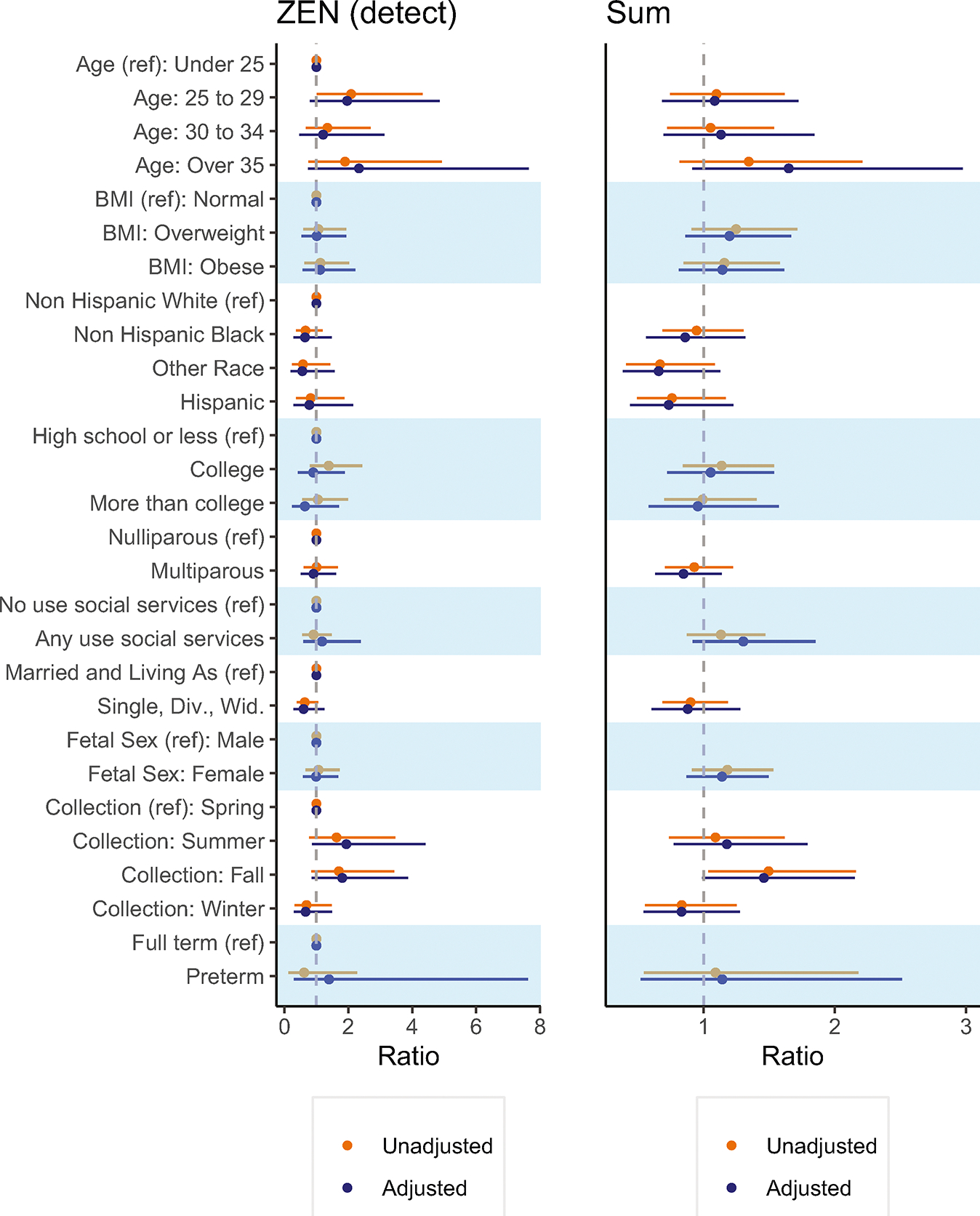
Ratio of placental mycoestrogen concentrations (ng/g) by sociodemographic, lifestyle, and perinatal characteristics of UPSIDE participants (*N* = 271). ^†^Ratios were calculated as the exponent of the beta coefficient. The mutually adjusted models (*n* = 270) were adjusted for all considered predictors (i.e., maternal age, early pregnancy BMI, race/ethnicity, parity, use of social services, marital status, fetal sex, season of urine collection. For models with ZEN, the response variable is binary (detect/non-detect), for models with sum of mycoestrogen analytes missing values were assigned LOD/√2. aZOL alpha-zearalenol, ZEN zearalenone, SUM sum of mycoestrogen analytes.

Analysis of individual predictors of placental mycoestrogens indicated a trend whereby higher early pregnancy BMI was associated with higher exposure (Fig. 3 and Supplementary Tables 9, 10). Non-Hispanic Black, and Hispanic participants tended to have lower ratios than Non-Hispanic White participants. Pregnancies with female fetuses also trended higher (Σmycoestrogens ratio 1.18; 95% CI: 0.91, 1.53). In unadjusted models, placentas collected in the fall had higher concentrations than the reference groups (Σmycoestrogens ratio 1.49; 95%CI: 1.03, 2.16). A sensitivity analysis including three placentas partially delivered and excluded from the primary analysis showed very similar results (Supplementary Table 11).

### Dietary exposure to mycoestrogens

**Fig. 4:**
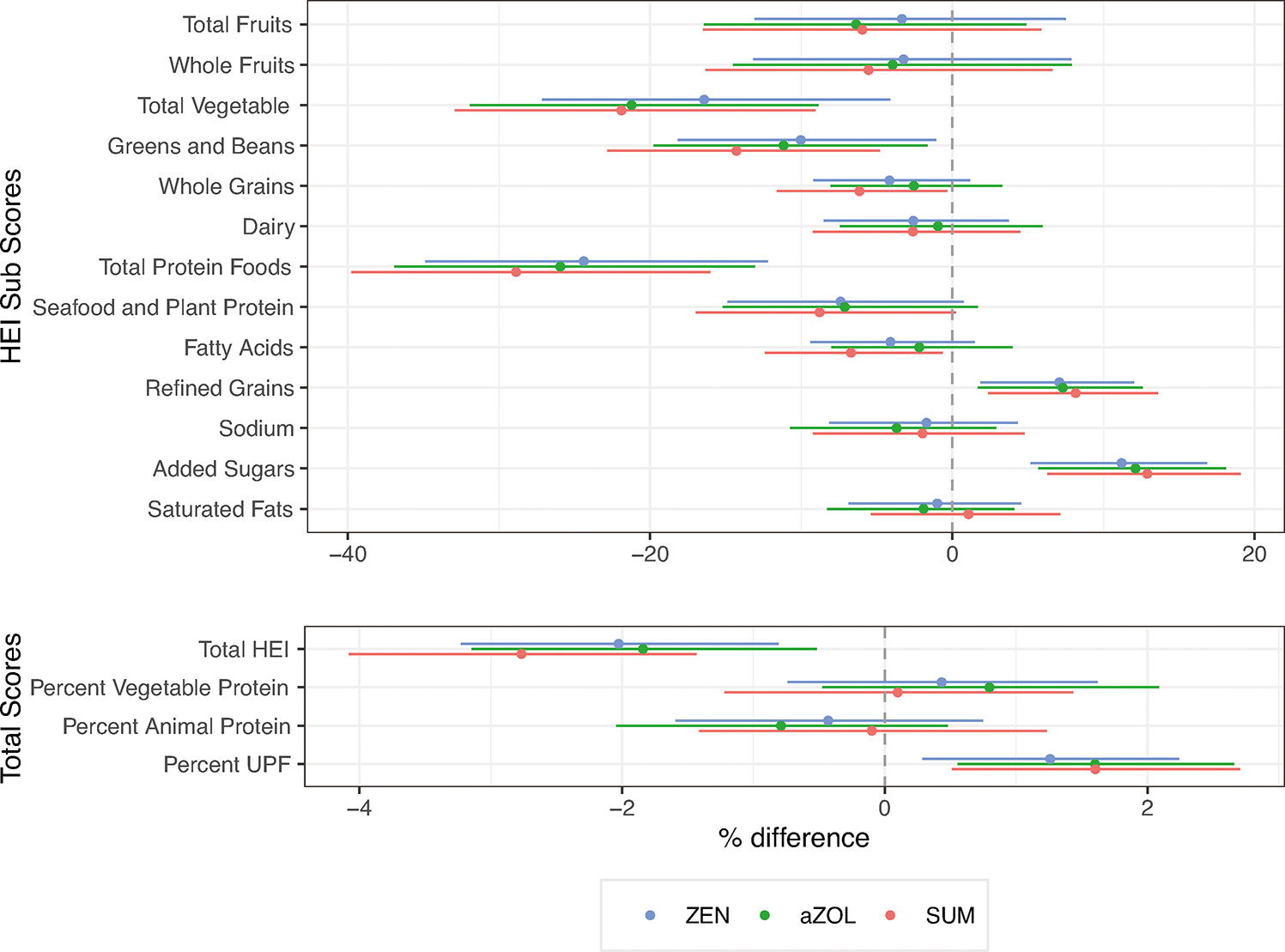
Urinary mycoestrogen concentrations (ng/ml) in relation to dietary parameters in UPSIDE participants (*N* = 172). ^†^To facilitate interpretation, the Healthy Eating Index moderation (refined grains, sodium, added sugars, and saturated fats) subscores are multiplied by −1 such that that higher scores indicate higher consumption. The maximum score for total fruit, whole fruit, vegetable, greens and beans, total protein, seafood, and plant protein, is 5, and for whole grains. dairy, fatty acids, refined grains, sodium, added sugars, and saturated fats it is 10. Dietary measures are averages of two dietary recalls connected in the second trimester. All mycoestrogen concentrations are specific gravity adjusted and missing values were assigned LOD/√2. aZOL alpha-zearalenol, ZEN zearalenone, SUM sum of mycoestrogen analytes.

In this cohort HEI-2015 scores were positively associated with vegetable protein intake and inversely to animal protein intake (Supplementary Table 12), UPF consumption was inversely related to vegetable, greens and beans, fruit and protein scores. UPSIDE participants with two dietary recalls in the second trimester (*n* = 172) had average HEI scores ranging from 27.3 to 92.3 (Supplementary Table 13). Unadjusted results are reported in Supplementary Table 13. In adjusted models, higher HEI scores were associated with lower 2nd trimester urinary mycoestrogen exposure (ZEN %Δ −2.03; 95%CI: −3.23, −0.81; α-ZOL %Δ−1.84; 95%CI: −3.15, −0.52; Σmycoestrogens %Δ −2.77; 95%CI: −4.08, −1.43) (Fig. 4 and Supplementary Table 14). Higher sub-scores for higher total protein and total vegetable intake were inversely related to urinary mycoestrogens (total protein ZEN %Δ −24.39; 95%CI: −34.89, −12.22; total vegetable ZEN %Δ −16.42; 95%CI: −27.15, −4.12). Higher sub-scores for refined grains and added sugars indicated positive associations with urinary mycoestrogens (refined grains ZEN %Δ 7.08; 95%CI: 1.86, 12.03 added sugars ZEN %Δ 11.20; 95%CI: 5.17, 16.85). Higher percentage of calories from UPF was associated with higher mycoestrogen exposure (ZEN %Δ 1.26; 95%CI: 0.29, 2.24; α-ZOL %Δ 1.60; 95%CI: 0.55, 2.66; Σmycoestrogens %Δ 1.60; 95%CI: 0.51, 2.71). In a sensitivity analysis, in models that were adjusted for total HEI score, sub-scores for vegetable and protein intake were inversely related to exposure, and refined grains and added sugar were positively related to mycoestrogen concentrations (Supplementary Table 15). In a post hoc analysis (Supplementary Table 16) examining diet quality by sociodemographic predictors BMI and parity, we found that HEI scores were higher in low BMI participants (low BMI $\overset{-}{x}=58.1$, high BMI $\overset{-}{x}=52.1$, *p* = 0.005) and UPF consumption was higher in high BMI participants (low BMI $\overset{-}{x}=51.1$, high BMI $\overset{-}{x}=56.21$, p = 0.055). There were not significant differences in diet quality by parity, though HEI-2015 trended lower in multiparous participants.

## DISCUSSION

In this study of mycoestrogen exposure in pregnancy, we detected measurable levels in 84% of placentas and nearly 100% of maternal urine samples. To the best of our knowledge, this is the first study to serially assess mycoestrogens in maternal urine and the placenta, alongside factors that may influence exposure levels, including detailed demographic, perinatal, and dietary information. We observed that maternal BMI and parity were associated with urinary concentrations, while season of sampling was associated with placental concentrations. The sociodemographic predictors observed are likely a proxy for differences in dietary patterns, for in this population we additionally found that lower HEI scores and higher consumption of UPF were associated with higher mycoestrogen concentrations. Samples collected later in pregnancy had higher mycoestrogen concentrations.

A small literature has examined mycoestrogen exposure during pregnancy [29, 30]. Of the two prior studies in pregnant people, concentrations in a population in Bangladesh (*n* = 20, median ZEN 0.185 ng/ml) were higher than those observed in our study, while levels in a small US sample (*n* = 30, mean ZEN 0.10 ng/ml) were lower than ours [29, 30]. Relative to other studies in North America, Σmycoestrogens were slightly lower than studies in peripubertal girls (*n* = 163, Σmycoestrogens = 0.32 ng/ml) [5]. These disparities may reflect differences in sample preparation (no deconjugation step) and/or age-related differences in diet and metabolism, as well as circulating endogenous estrogens. Endogenous circulating estrogens may impact the conjugation of mycoestrogens; in this analysis we found that 83% of urinary mycoestrogens were conjugated. Similar prevalence of exposure was observed in US post-menopausal women (*n* = 48), where circulating serum Σmycoestrogens were detected in all samples [54]. We observed several patterns that were consistent with the larger international biomonitoring literature in non-pregnant populations including: (1) high prevalence of exposure, (2) seasonal variation in exposure, (3) metabolite profiles (aZOL > ZEN > bZOL), (4) sex (in this study: fetal sex) differences in concentrations, (5) high proportion of conjugated metabolites [7, 29, 59] (Supplementary Table 17). Additionally, we identified a pattern of increasing mycoestrogen concentrations across pregnancy. Several studies have reported differences in concentrations across pregnancy for other non-persistent endocrine disrupting chemicals including phenols, organophosphate ester flame retardants, and plasticizers [60–62]. Similar to other chemicals, differences in mycoestrogens concentrations, though significant, were small in magnitude [61].

Demographic predictors of mycoestrogen exposure have not been investigated in pregnant people. We observed higher exposure in women with a BMI indicating overweight relative to normal BMI, though associations in women with obesity were inconsistent. In our primary analysis, pre-pregnancy BMI indicative of obesity was not a significant predictor, but obesity was a significant predictor (adjusted ratio 1.25 95%CI 1.02, 1.52) in a sensitivity analysis where we did not replace aZOL values below LOD. One prior study examining serum mycoestrogens in relation to BMI in post-menopausal women reported a negative correlation between BMI and serum total ZEN, and in contrast, positive correlations with unconjugated ZEN and unconjugated ZEN metabolites [54]. Higher levels in overweight participants could reflect differences in dietary patterns and overall intake, or metabolic differences; or these findings could be in part due to the matrix used in that analysis (serum). We additionally explored associations between mycoestrogens and race and ethnicity in unadjusted and adjusted models. In this study, we did not find race and ethnicity were significant predictors of urine or placental concentrations. Here a null result may reflect the relatively small sample size and limited geography and diversity of this cohort. Ideally, trends of exposure across racial and ethnic groups will be investigated in a larger and more diverse cohort. Race and ethnicity are important to consider as predictors because they are a proxy for structural racism which may impact dietary quality [46]; and neighborhood education levels is linked to consumption of fruits and vegetables [63]. Parity was the only considered factor, in addition to pre-pregnancy BMI, that predicted higher urinary concentrations after adjustment. Parity is linked to household size and food budget per household member, which studies show determines nutritional quality of food purchased and consumed in the US and around the world [64, 65]; these facts may explain the association of multiparity with higher exposure. We note that estimates showed slight variation depending on which mycoestrogen measure was the outcome (e.g., parity ZEN ratio 1.03 95% CI: 0.98, 1.09; aZOL ratio 1.06 95%CI: 1.00, 1.12; Σmycoestrogens 1.03 95% CI 0.98, 1.09). This variation could be due to differences in exposure, metabolism, or an undetermined factor.

Abundant research details mycoestrogen contamination of food, while fewer studies link dietary sources to biomarkers of exposure in humans. In the US contamination of ZEN in raw corn is reported as 39.8 ± 79.7 μg/kg, with one study showing that over 80% of corn samples were contaminated [66]. Of corn by-products produced by wet-milling the highest levels of contamination were in corn gluten (329.8 ± 237.6) and corn germ (298.9 ± 245.0 μg/kg), which are common components of processed and UPF foods [66]. The US does not impose guidance on ZEN in foodstuffs, however in the EU there are limits on that amount in unprocessed cereals (100 μg/kg), unprocessed corn (350 μg/kg), refined corn oil (400 μg/kg), breakfast cereals and snacks (100 μg/kg), and processed corn-based foods for infants and young children (20 μg/kg). Korea sets even lower guidance levels [66]. Multiple studies report that corn oil is also frequently contaminated at much higher levels than raw or milled corn [67, 68]. Dietary sources of mycoestrogen exposure may vary with cultural food consumption patterns and geography. Studies using FFQs or dietary recalls in the US and Portugal have reported associations between urinary mycoestrogen concentrations and consumption of corn, meat, and dairy [69]. In rural China, a study which linked duplicate dietary recalls to human biomonitoring estimated that wheat contributed 80% of the daily intake of ZEN [8]. Finally, processed food (cookies, biscuits) intake has been associated with mycoestrogen levels in lactating women and French children [70, 71].

In this study, we assessed diet in two main ways: (1) HEI and (2) % of calories from UPF. A strength of these measures is that they are standardized to caloric intake, thus associations with particular foods do not reflect simply overall greater food consumption. Importantly, UPF consumption has increased over the past two decades to nearly 60% of calories consumed, reportedly across demographic strata [72]. Prior research in the US showing that HEI varies by race and ethnicity [73]. Other markers of socioeconomic status, such as education could be related to household income, which influences food quality. For example, higher incomes are associated with higher quality diets and higher HEI scores [65]. We additionally considered overall energy intake, and percent of protein from animal or vegetable sources, but did not observe significant associations with those dietary measures. In this study we found that higher BMI participants had higher mycoestogens and lower diet quality.

Our study has notable strengths. We are the first to report serial assessment of urinary mycoestrogen concentrations in pregnancy. Our robust analytic protocol leverages high resolution mass spectrometry to attain lower limits of detection than prior studies. We used data from a well-characterized pregnancy cohort, with detailed demographic information and duplicate high-quality dietary recalls. We conducted multiple sensitivity analysis. At the same time, limitations are noted. In this study, creatinine concentrations were not assessed, therefore we could not consider alternative methods for correcting for urine dilution. The chemical structure of the synthetic xenobiotic ZER is indistinguishable from the naturally occurring ZEN metabolite αZAL, therefore we cannot determine the relative contributions from natural versus artificial sources. Additionally, though we report on placental mycoestrogen concentrations, further research is needed on ZEN concentration and conjugation in cord blood, neonate meconium, and urine, to more comprehensively characterize fetal exposures. In this study, we averaged two dietary recalls collected in the second trimester to estimate usual intake, for a subset of UPSIDE participants. The recalls were not temporally matched to exposure assessment, and for some participants, one of the recalls may have occured prior to urine collection.

Mycotoxin contamination of food has impacted humans since the dawn of agriculture. The industrialization of food, particularly commodity crops such as corn, soy, and wheat, has the potential to either limit exposure to mycoestrogens through regulation and testing of food products, but by the same token, also has the potential to concentrate mycotoxin exposure through storage, handling, and processing. Here, we find that exposure to this estrogenic mycotoxin is nearly ubiquitous during pregnancy, when developmental programming is sensitive to environmental estrogens [74]. Climate change may alter geographical patterns of mycotoxin food contamination, further highlighting the need for testing and regulation. We found that diets lower in protein and vegetables, and higher in added sugars and UPF were associated with higher mycoestrogen exposure. Demographic factors aside from BMI were not strongly linked to exposure in this cohort, but that requires further study because they are linked to socioeconomic status, and may reflect disparities in access to fresh foods [46]. Given the experimental evidence that mycoestrogens impact pregnancy and child health further research is warranted as to the source of exposure across the lifespan and impacts on pregnancy. Monitoring and regulations of mycotoxins and synthetic derivatives in food have the potential to reduce exposure and reduce potential health impacts.

## Supplementary Material

supplementary materials

## Figures and Tables

**Table 1.** Characteristics of UPSIDE participants contributing to this analysis (*N* = 292)^a^.

Continuous Variables	Mean (SD)
Kilocalories per day^b^	2161.6 (321.3)
Percent of calories from UPF^b^	53.8 (17.0)
Healthy Eating Index^b^	54.9 (13.8)
% protein from vegetable source^b^	38.4 (14.2)
% protein from animal source^b^	61.6 (14.2)
*Categorical Variables*	*N (%)*
Maternal age (years)	
<25	52 (17.8)
25–29	98 (33.6)
30–34	108 (37.0)
≥35	34 (11.6)
Early pregnancy BMI (kg/m^2^)	
<25	127 (43.5)
25–29	77 (26.4)
>30	88 (30.1)
Full term	277 (94.9)
Ethnicity/race	
Hispanic	31 (10.6)
Non-Hispanic White	168 (57.5)
Non-Hispanic Black	69 (23.6)
Asian, Pacific Islander, Mixed Race, Other	24 (8.2)
Education	
Less than high school/high school	108 (37.0)
Some college/college	112 (38.4)
Post-secondary	71 (24.3)
Nulliparous	99 (33.9)
Use of social services in pregnancy (any)	162 (55.5)
Married/Living as Married	171 (58.6)
Infant Sex (male)	143 (49.0)
Urine sample collection season	
Spring	231 (29.8)
Summer	226 (29.2)
Fall	147 (19.0)
Winter	170 (22.0)
Placenta sample collection season	
Spring	50 (18.4)
Summer	67 (24.7)
Fall	96 (35.4)
Winter	58 (21.4)

*BMI* body mass index, *UPF* ultra-processed foods.

a The *N* for urine samples is 258; the *N* for placenta samples is 271. There was missing data for education (*n* = 1) and marital status (*n* = 7).

b The *N* for dietary variables is 172 and are averages from two dietary recalls in the second trimester.

**Table 2.** Distribution of mycoestrogens in urine (ng/ml) and placenta (ng/g) in the UPSIDE cohort.

Analyte	Matrix	N	LOD	%>LOD	GM (GSD)^a^	Percentiles				Max
						25th	50th	75th	95th	
bZAL	Urine	774	0.018	30.4	—	<LOD	<LOD	0.028	0.105	1.713
bZAL	Placenta	271	0.006	13.7	—	<LOD	<LOD	<LOD	0.011	0.493
bZOL	Urine	774	0.028	47.8	—	<LOD	<LOD	0.072	0.248	1.545
bZOL	Placenta	271	0.007	8.1	—	<LOD	<LOD	<LOD	0.004	0.06
aZAL	Urine	774	0.015	21.4	—	<LOD	<LOD	<LOD	0.055	0.823
aZAL	Placenta	271	0.005	17	—	<LOD	<LOD	<LOD	0.009	0.771
aZOL	Urine	774	0.018	83.9	0.136 (2.917)	0.050	0.138	0.271	0.822	4.135
aZOL	Placenta	271	0.011	26.9	—	<LOD	<LOD	0.003	0.009	1.035
ZAN	Urine	774	0.010	16.1	—	<LOD	<LOD	<LOD	0.018	0.414
ZAN	Placenta	271	0.006	16.2	—	<LOD	<LOD	<LOD	0.006	0.816
ZEN	Urine	774	0.011	95.6	0.127 (2.743)	0.066	0.123	0.24	0.682	3.7
ZEN	Placenta	271	0.008	59.4	—	<LOD	0.005	0.013	0.027	0.203
SUM	Urine	774	0.010	97	0.295 (3.325)	0.159	0.323	0.617	1.662	9.443
SUM	Placenta	271	0.005	84.1	0.010 (2.975)	0.003	0.012	0.019	0.047	2.094

The geometric mean was calculated only for analytes detected in more than 75% of specimens and concentrations below LOD were replaced with LOD/sqrt [2].

a The data reported for urine concentrations represents samples from all three trimesters.

*aZAL* alpha-zearalanol, *aZOL* alpha-zearalenol, *bZAL* beta-zearalanol, *bZOL* beta-zearalenol, *GM* geometric mean, *GSD* geometric standard deviation, *Max* maximum, *ZAN* zearalanone, *ZEN* zearalenone, *SUM* sum of mycoestrogen analytes.

## Data Availability

The datasets generated and analyzed in the current study are available from the corresponding author on reasonable request.
