# Supplementary material for: Sociodemographic and dietary predictors of maternal and placental mycoestrogen concentrations in a US pregnancy cohort
Source: J Expo Sci Environ Epidemiol. 2024 Oct 4;35(3):382–92. doi: 10.1038/s41370-024-00722-6 (PMC11968447; doi:10.1038/s41370-024-00722-6)
Supplement: Supplementary file 1 — Supplementary Methods [file 41370_2024_722_MOESM1_ESM.pdf]

Supplementary Methods. Mycoestrogen quantitation by UPLC-MS/MS.

An autosampler injected 10 µL into the UPLC-MS/MS. To achieve separation of the analytes, the solvent flow rate was 0.5 ml/minute. The initial gradient conditions started at: solvent A 50% water; solvent B 25% methanol; solvent C 25% acetonitrile for 0.5 minutes. At 3 minutes the linear gradient was set to 20% A, 40% each B and C, then at 5 minutes the linear gradient was set to 5% A, 47.5% each B and C and held for 11 minutes. Following this, the gradient returned to initial conditions. Each sample's total run time was 16 minutes. The elution time (minutes) for each analyte was: ZEN: 8.44, α-ZOL 7.92, β-ZOL: 7.41, α-ZAL: 7.79, ZEN: 8.39, β-ZOL 7.32, ZEN d_6: 8.45. The average recovery for individual analytes in urine ranged from 0.90 to 1.03; and 0.68 to 0.80 for placenta. Daily LOD was used to determine the validity of measurements. The average daily LOD for each analyte in urine was: α-ZOL (0.0025 ng/ml), β-ZOL (0.0040 ng/ml), ZER (0.0021 ng/ml), β-ZAL (0.0025 ng/ml), ZAN (0.0014 ng/ml), and ZEN (0.0011 ng/ml). Standard addition method was used to determine concentrations of experimental samples due to background levels in calibration curve samples. Samples that were beyond the linear range of the standard were diluted and reanalyzed to confirm

Supplementary Figure 1. UPSIDE participants contributing data to the current analysis.

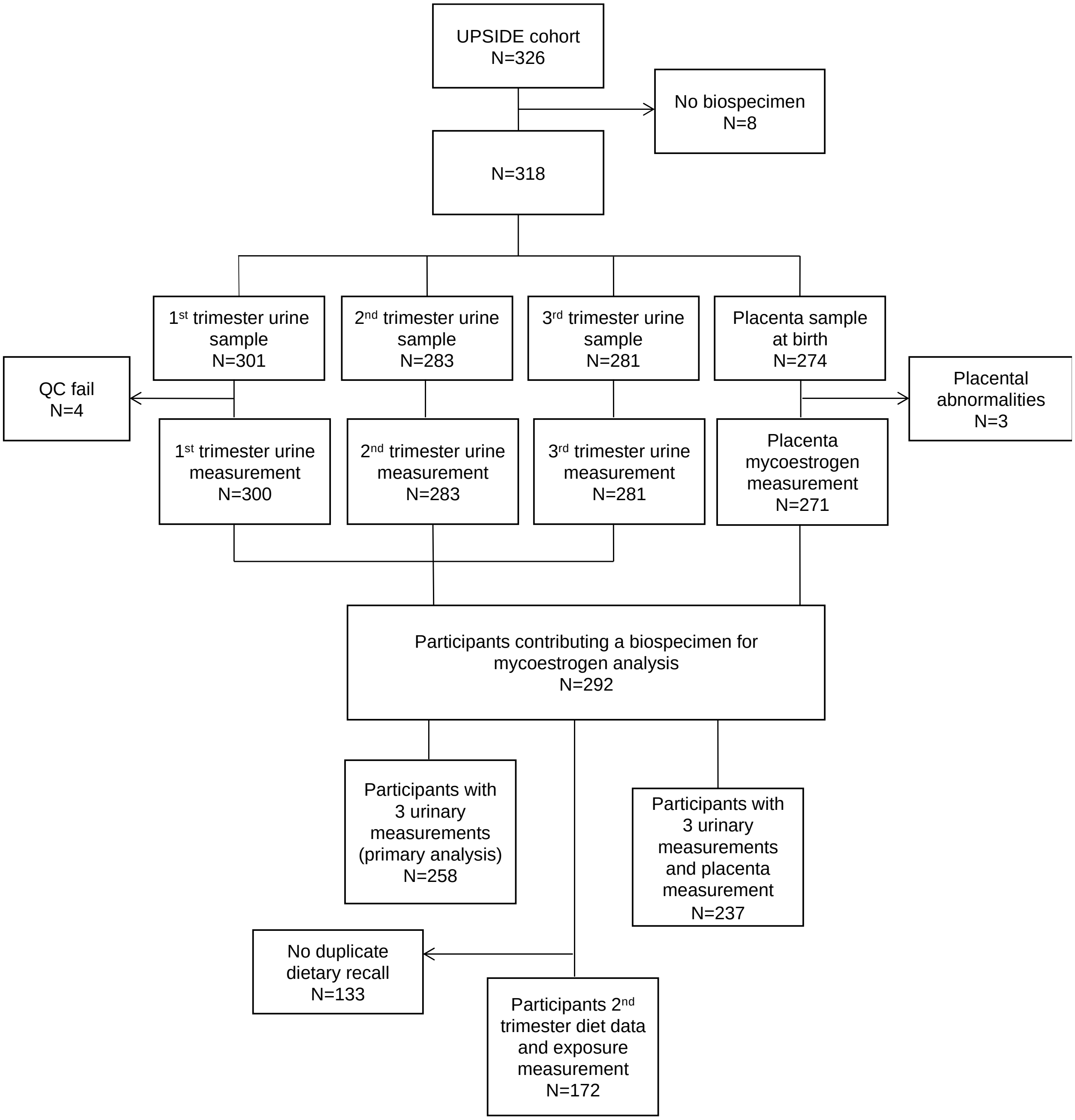

Supplementary Table 1. P-value for chi-square test of independence of categorical predictor variables

	Maternal Age	*Education*	*Race and Ethnicity*	*BMI*	*Parity*	*Infant Sex*	*Marital Status*	*Use of social services*
Maternal Age	-	-	-	-	-	-	-	-
Education	**<0.01**	-	-	-	-	-	-	-
Race and Ethnicity	**<0.01**	**<0.01**	-	-	-	-	-	-
BMI	0.11	**<0.01**	**<0.01**	-	-	-	-	-
Parity	**0.02**	0.25	0.09	0.20	-	-	-	-
Infant Sex	0.74	0.56	0.32	0.76	1	-	-	-
Marital Status	**<0.01**	**<0.01**	**<0.01**	**<0.01**	0.77	0.64	-	-
Support	**<0.01**	**<0.01**	**<0.01**	**<0.01**	0.05	0.52	**<0.01**	-
Preterm	0.15	**<0.01**	**<0.01**	0.15	0.66	0.26	0.20	0.22

†P-values less that 0.05 are bold.
Abbreviations: BMI: body mass index

Supplementary Figure 2. Comparison of free versus conjugated zearalenone (ng/ml) in maternal urine specimens from the UPSIDE cohort (n=30)

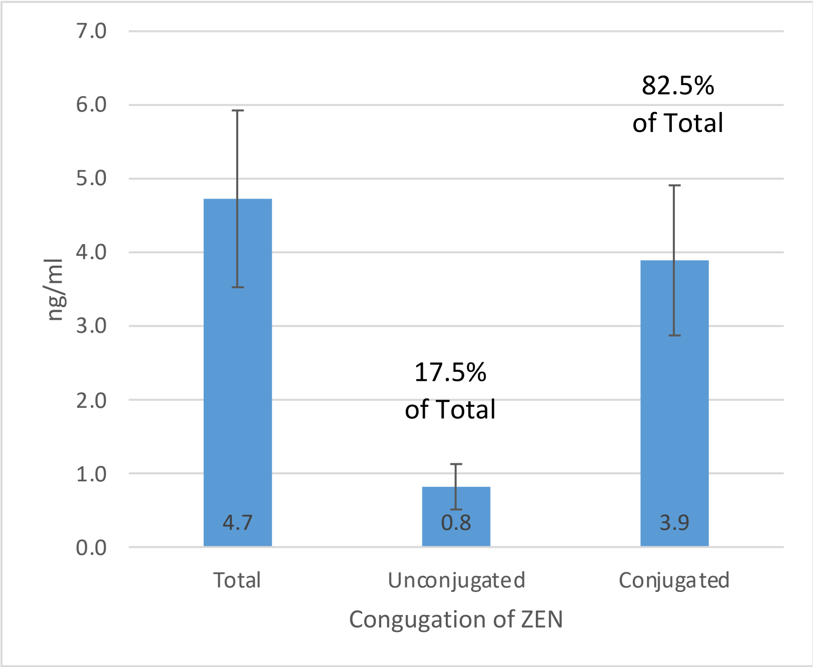

Abbreviations: Free: unconjugated before enzymatic digestion, ZEN: zearalenone.

Supplementary Table 2. Concentration and intraclass correlation of urinary (ng/ml) and placental (ng/g) mycoestrogens in the UPSIDE cohort (n=271-300)

		N	%> LOD	GM†	GSD†	P25	P50	P75	P95	Max	ICC (CI)‡
**bZAL**	1st	300	24.9	-	-	<LOD	<LOD	<LOD	0.0802	0.3405	-
	2nd	283	44.9	-	-	<LOD	<LOD	0.0404	0.1044	1.7126	
	3rd	281	22.4	-	-	<LOD	<LOD	<LOD	0.1342	1.6674	
	Placenta	271	13.7	-	-	<LOD	<LOD	<LOD	0.0110	0.4930	-
**bZOL**	1st	300	46.5	-	-	<LOD	<LOD	0.0529	0.1670	0.7423	-
	2nd	283	51.6	-	-	<LOD	<LOD	0.0713	0.2246	1.5454	
	3rd	281	45.2	-	-	<LOD	<LOD	0.0882	0.3108	2.2943	
	Placenta	271	8.1	-	-	<LOD	<LOD	<LOD	0.0043	0.0595	-
**aZAL**	1st	300	27.2	-	-	<LOD	<LOD	0.0012	0.0973	0.8233	-
	2nd	283	7.4	-	-	<LOD	<LOD	<LOD	0.0020	0.1315	
	3rd	281	27.4	-	-	<LOD	<LOD	<LOD	0.0427	0.8021	
	Placenta	271	17.0			<LOD	<LOD	<LOD	0.0092	0.7707	-
**aZOL**	1st	300	76.4	0.0924	2.5924	<LOD	0.0750	0.1556	0.4602	2.7145	0.22 (0.15, 0.30)
	2nd	283	88.0	0.1344	2.7863	0.0327	0.1142	0.2262	0.6745	2.6206	
	3rd	281	90.4	0.2251	2.9022	0.0754	0.2011	0.3816	1.0539	5.6204	
	Placenta	271	26.9	-	-	<LOD	<LOD	0.0027	0.0090	1.0345	-
**ZAN**	1st	300	18.9	-	-	<LOD	<LOD	<LOD	0.0178	0.3657	-
	2nd	283	19.8	-	-	<LOD	<LOD	<LOD	0.0124	0.1173	
	3rd	281	8.9	-	-	<LOD	<LOD	<LOD	0.0183	0.4138	
	Placenta	271	16.2	-	-	<LOD	<LOD	<LOD	0.0057	0.8156	-
**ZEN**	1st	300	94.0	0.1035	2.6304	0.0494	0.0939	0.1742	0.6364	1.6741	0.20 (0.12, 0.28)
	2nd	283	99.3	0.1139	2.5665	0.0446	0.1009	0.1965	0.5127	2.9657	
	3rd	281	93.2	0.1860	3.0558	0.0702	0.1603	0.3146	0.8267	10.9702	
	Placenta	271	59.4	0.0058	2.49	<LOD	0.0049	0.0126	0.0267	0.2025	-
**SUM**	1st	300	95.0	0.2704	3.5556	0.0929	0.2061	0.4127	1.3523	5.1933	0.16 (0.09, 0.24)
	2nd	283	99.3	0.3703	3.1813	0.1251	0.2706	0.5151	1.4597	7.6607	
	3rd	281	97.5	0.5913	3.2541	0.1801	0.4228	0.8212	2.2404	20.5809	
	Placenta	271	84.1	0.0098	2.98	0.0033	0.0116	0.0186	0.0475	2.0937	-

†Geometric means and standard deviations were calculated for analytes with detection frequencies >75% in urine samples and >50% in placental samples; the values below LOD were imputed with LOD/√2.
‡ ICC was calculated for analytes with detection frequencies >75%; the values below LOD were replaced with LOD/√2, values were log transformed.
Abbreviations: bZAL: beta-zearalanol, bZOL: beta-zearalenol, aZAL: alpha-zearalanol, aZOL: alpha-zearalenol, ZAN: zearalanone, ZEN: zearalenone, Sum: sum of metabolite concentrations. 1st: first trimester, 2nd: second trimester, 3rd: third trimester, N: sample number, LOD: limit of detection, GM: geometric mean, GSD: geometric standard deviation. ICC: intraclass correlation, CI: confidence interval.

Supplementary Table 3. Spearman correlation between log-transformed mycoestrogen concentrations and dietary parameters.

	**ZEN (Urine)**	**aZOL (Urine)**	**SUM (Urine)**	**SUM (Placenta)**	**Percent UPF**	**HEI**	**kcal/day**
**ZEN (Urine)**	**1**	**0.79**	**0.89**	0	**0.11**	-0.09	-0.07
**aZOL (Urine)**	**0.79**	**1**	**0.86**	0.03	**0.12**	**-0.11**	-0.04
**SUM (Urine)**	**0.89**	**0.86**	**1**	0.02	**0.14**	**-0.17**	-0.06
**SUM (Placenta)**	0	0.03	0.02	1	0.03	-0.04	-0.07
**Percent UPF**	**0.11**	**0.11**	**0.14**	0.03	1	**-0.3**	**0.1**
**HEI**	-0.09	**-0.11**	**-0.17**	-0.04	**-0.3**	1	-0.06
**kcal/day**	-0.07	-0.04	-0.06	-0.07	**0.1**	-0.06	1

[†]The N for urine measurements is 258 (3 measurements per person), placental measurement is 271, dietary measurements is 172. Bold text indicates significance at $p<0.05$. All mycoestrogen concentrations are specific gravity adjusted and missing values were assigned LOD/√2.
Abbreviations: aZOL: alpha-zearalenol, bZAL: ZEN: zearalenone, HEI: Healthy Eating Index, UPF: ultraprocessed foods, SUM: sum of mycoestrogen analytes.

Supplementary Figure 3. Spearman correlation between log-transformed mycoestrogen metabolites concentrations across pregnancy (N=237)†

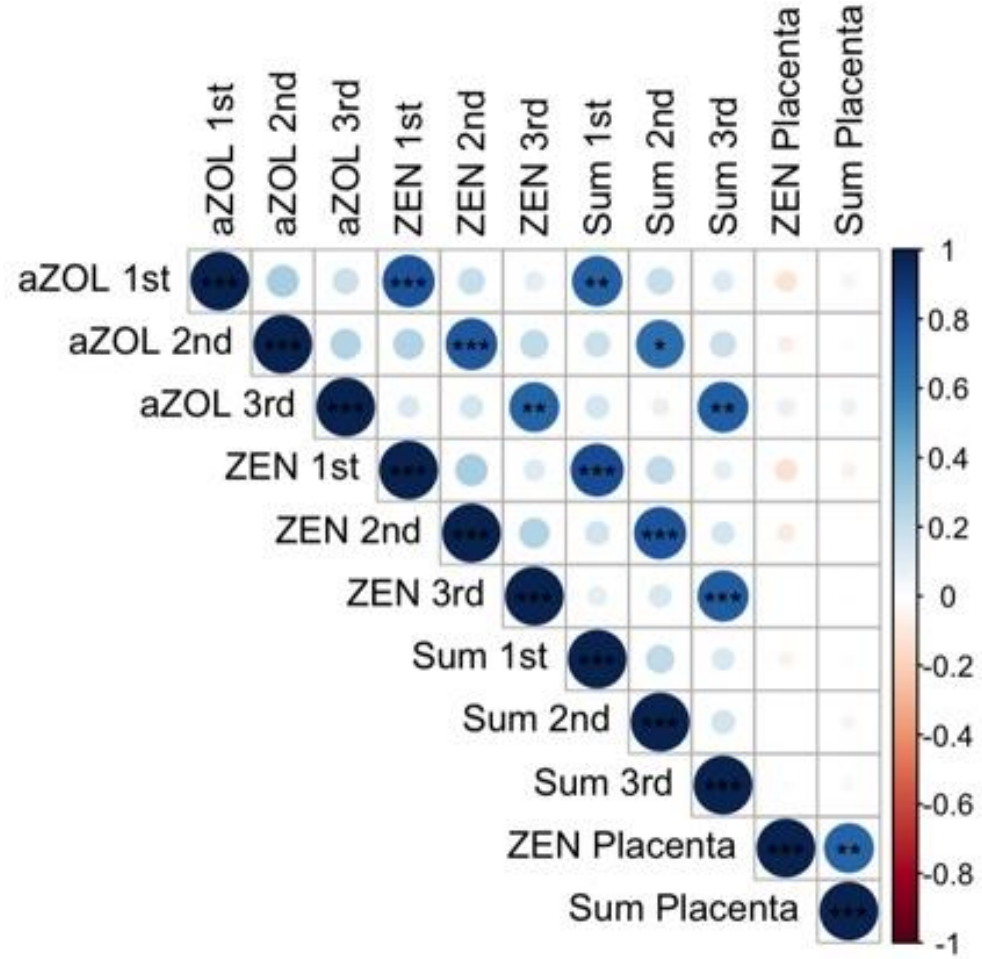

†Values below LOD were replaced with LOD/√2, values were log transformed. Significance indicated at *p<0.05, **p<0.01, ***p<0.001.

Abbreviations: aZOL: alpha-zearalenol, ZEN: zearalenone, Sum: sum of metabolite concentrations. 1st: first trimester, 2nd: second trimester, 3rd: third trimester.

Supplementary Table 4. Unadjusted ratio and ß estimates of log-transformed mycoestrogen concentrations (ng/ml) by sociodemographic, lifestyle, and perinatal characteristics of UPSIDE participants (N=258) contributing 3 urine samples.[†]

	ZEN		*aZOL*		*SUM*	
	Ratio (95% CI)†	*ß (95% CI)*	*Ratio (95% CI)†*	*ß (95% CI)*	*Ratio (95% CI)†*	*ß (95% CI)*
**Maternal Age (years)**						
**Under 25**	Ref	Ref	Ref	Ref	Ref	Ref
**25-29**	0.98 (0.91, 1.06)	-0.02 (-0.09, 0.06)	0.95 (0.87, 1.03)	-0.06 (-0.14, 0.02)	0.93 (0.77, 1.12)	
**30-34**	0.99 (0.92, 1.06)	-0.01 (-0.09, 0.06)	0.94 (0.87, 1.02)	-0.06 (-0.14, 0.02)	0.92 (0.76, 1.11)	
**Over 35**	1.00 (0.92, 1.10)	0.00 (-0.09, 0.09)	0.95 (0.86, 1.05)	-0.05 (-0.15, 0.05)	0.96 (0.76, 1.21)	
**Early Pregnancy BMI**						
**Normal**	Ref	Ref	Ref	Ref	Ref	Ref
**Overweight**	**1.07 (1.01, 1.13)**	**0.07 (0.01, 0.12)**	**1.09 (1.02, 1.15)**	**0.08 (0.02, 0.14)**	**1.19 (1.03, 1.37)**	
**Obese**	0.99 (0.94, 1.05)	-0.01 (-0.06, 0.05)	**1.08 (1.02, 1.15)**	**0.08 (0.02, 0.14)**	1.09 (0.95, 1.24)	
**Ethnicity/Race**						
**Non-Hispanic White**	Ref	Ref	Ref	Ref	Ref	Ref
**Non-Hispanic Black**	1.01 (0.95, 1.07)	0.01 (-0.05, 0.07)	**1.06 (1.00, 1.13)**	**0.06 (0.00, 0.12)**	1.10 (0.95, 1.27)	
**Other races**	0.96 (0.88, 1.04)	-0.04 (-0.13, 0.04)	0.95 (0.87, 1.05)	-0.05 (-0.14, 0.05)	0.91 (0.73, 1.13)	
**Hispanic**	0.99 (0.91, 1.07)	-0.01 (-0.09, 0.07)	1.06 (0.97, 1.15)	0.06 (-0.03, 0.14)	1.07 (0.87, 1.31)	
**Education**						
**Less than high school/high school**	Ref	Ref	Ref	Ref	Ref	Ref
**Some college/college**	1.01 (0.96, 1.07)	0.01 (-0.04, 0.07)	0.98 (0.92, 1.04)	-0.02 (-0.08, 0.04)	0.98 (0.86, 1.13)	
**Post-secondary**	0.98 (0.93, 1.04)	-0.02 (-0.08, 0.04)	**0.93 (0.87, 0.99)**	-0.07 (-0.14, -0.01)	0.88 (0.76, 1.02)	
**Parity**						
**Nulliparous**	Ref	Ref	Ref	Ref	Ref	Ref
**Multiparous**	1.03 (0.98, 1.08)	0.03 (-0.02, 0.08)	**1.06 (1.00, 1.11)**	**0.06 (0.00, 0.11)**	**1.13 (1.00, 1.28)**	
**Support**						
**None**	Ref	Ref	Ref	Ref	Ref	Ref
**Any**	1.00 (0.96, 1.05)	0.00 (-0.05, 0.05)	1.04 (0.99, 1.09)	0.04 (-0.01, 0.09)	1.08 (0.96, 1.21)	
**Marital Status**						
**Married/Living as Married**	Ref	Ref	Ref	Ref	Ref	Ref
**Single/Divorced/Widowed/Separated**	1.00 (0.96, 1.05)	0.00 (-0.04, 0.05)	**1.06 (1.00, 1.11)**	0.05 (0.00, 0.11)	1.08 (0.96, 1.21)	
**Fetal Sex**						
**Male**	Ref	Ref	Ref	Ref	Ref	Ref
**Female**	1.03 (0.99, 1.08)	0.03 (-0.01, 0.08)	1.03 (0.98, 1.08)	0.03 (-0.02, 0.08)	1.10 (0.98, 1.24)	
**Season**						
**March, April, May**	Ref	Ref	Ref	Ref	Ref	Ref
**June, July, August**	1.03 (0.97, 1.09)	0.03 (-0.03, 0.08)	1.04 (0.98, 1.10)	0.04 (-0.02, 0.10)	1.06 (0.93, 1.22)	
**September, October, November**	1.02 (0.96, 1.09)	0.02 (-0.04, 0.09)	1.01 (0.94, 1.08)	0.01 (-0.06, 0.08)	1.02 (0.87, 1.20)	
**December, January, February**	0.97 (0.92, 1.03)	-0.03 (-0.09, 0.03)	0.93 (0.88, 1.00)	-0.07 (-0.13, 0.00)	0.90 (0.77, 1.04)	
**Sample collection**						

**1st trimester**	Ref	Ref	Ref	Ref	Ref	Ref
**2nd trimester**	1.02 (0.97, 1.07)	0.02 (-0.03, 0.07)	**1.08 (1.02, 1.14)**	**0.08 (0.02, 0.13)**	**1.14 (1.01, 1.29)**	
**3rd trimester**	**1.16 (1.10, 1.22)**	**0.14 (0.09, 0.20)**	**1.25 (1.18, 1.32)**	**0.22 (0.17, 0.27)**	**1.49 (1.32, 1.69)**	

[†]Ratios were calculated as the exponent of the beta coefficient of a model where the mycoestrogen concentration was the response variable. All mycoestrogen concentrations are specific gravity adjusted and missing values were assigned LOD/$\sqrt{2}$. Bold text indicates significance at $p<0.05$.

Abbreviations: aZOL: alpha-zearalenol, ZEN: zearalenone, SUM: sum of mycoestrogen analytes.

Supplementary Table 5. Adjusted ratio and ß estimates of log-transformed serially assessed urinary mycoestrogen concentrations (ng/ml) by sociodemographic, lifestyle, and perinatal characteristics of UPSIDE participants (N=258) contributing 3 urine samples. †

	ZEN		***aZOL***		***SUM***	
	Ratio (95% CI)†	*ß (95% CI)*	*Ratio (95% CI)†*	*ß (95% CI)*	*Ratio (95% CI)†*	*ß (95% CI)*
**Maternal Age (years)**						
**Under 25**	Ref	Ref	Ref	Ref	Ref	Ref
**25-29**	0.97 (0.89, 1.05)	-0.03 (-0.11, 0.05)	0.93 (0.85, 1.01)	-0.08 (-0.16, 0.01)	0.97 (0.89, 1.05)	-0.03 (-0.11, 0.05)
**30-34**	0.97 (0.89, 1.06)	-0.03 (-0.12, 0.05)	0.93 (0.85, 1.02)	-0.07 (-0.16, 0.02)	0.97 (0.89, 1.06)	-0.03 (-0.12, 0.05)
**Over 35**	0.99 (0.90, 1.100	-0.01 (-0.11, 0.09)	0.93 (0.84, 1.04)	-0.07 (-0.17, 0.04)	0.99 (0.90, 1.10)	-0.01 (-0.11, 0.09)
**Early Pregnancy BMI**						
**Normal**	Ref	Ref	Ref	Ref	Ref	Ref
**Overweight**	**1.08 (1.01, 1.14)**	**0.07 (0.02, 0.13)**	**1.09 (1.02, 1.15)**	**0.08 (0.02, 0.14)**	**1.08 (1.02, 1.14)**	**0.07 (0.02, 0.13)**
**Obese**	0.98 (0.93, 1.04)	-0.02 (-0.07, 0.04)	1.05 (0.99, 1.12)	0.05 (-0.01, 0.11)	0.98 (0.93, 1.04)	-0.02 (-0.07, 0.04)
**Ethnicity/Race**						
**Non-Hispanic White**	Ref	Ref	Ref	Ref	Ref	Ref
**Non-Hispanic Black**	1.01 (0.94, 1.09)	0.01 (-0.07, 0.08)	1.03 (0.95, 1.11)	0.03 (-0.05, 0.11)	1.01 (0.94, 1.09)	0.01 (-0.07, 0.08)
**Other Races**	0.98 (0.89, 1.07)	-0.02 (-0.12, 0.07)	0.96 (0.87, 1.06)	-0.04 (-0.14, 0.06)	0.98 (0.89, 1.07)	-0.02 (-0.12, 0.07)
**Hispanic**	0.99 (0.90, 1.08)	-0.01 (-0.10, 0.08)	1.02 (0.93, 1.12)	0.02 (-0.07, 0.12)	0.99 (0.90, 1.08)	-0.01 (-0.10, 0.08)
**Education**						
**Less than high school/high school**	Ref	Ref	Ref	Ref	Ref	Ref
**Some college/college**	1.02 (0.96, 1.09)	0.02 (-0.04, 0.09)	1.03 (0.96, 1.10)	0.03 (-0.04, 0.09)	1.02 (0.96, 1.09)	0.02 (-0.04, 0.09)
**Post-secondary**	0.98 (0.91, 1.07)	-0.02 (-0.10, 0.07)	1.00 (0.91, 1.09)	0.00 (-0.09, 0.08)	0.98 (0.91, 1.07)	-0.02 (-0.10, 0.07)
**Parity**						
**Nulliparous**	Ref	Ref	Ref	Ref	Ref	Ref
**Multiparous**	1.03 (0.98, 1.09)	0.03 (-0.02, 0.08)	**1.06 (1.00, 1.12)**	**0.06 (0.00, 0.11)**	1.03 (0.98, 1.09)	0.03 (-0.02, 0.08)
**Use of Support**						
**None**	Ref	Ref	Ref	Ref	Ref	Ref
**Any**	0.98 (0.93, 1.04)	-0.02 (-0.08, 0.04)	0.98 (0.92, 1.04)	-0.02 (-0.09, 0.04)	0.98 (0.93, 1.04)	-0.02 (-0.08, 0.04)
**Marital Status**						
**Married/Living as Married**	Ref	Ref	Ref	Ref	Ref	Ref
**Single/Divorced/Widowed/Separated**	1.01 (0.94, 1.07)	0.01 (-0.06, 0.07)	1.03 (0.96, 1.10)	0.03 (-0.04, 0.10)	1.01 (0.94, 1.07)	0.01 (-0.06, 0.07)
**Fetal Sex**						
**Male**	Ref	Ref	Ref	Ref	Ref	Ref
**Female**	1.04 (0.99, 1.09)	0.04 (-0.01, 0.08)	1.04 (0.99, 1.09)	0.04 (-0.01, 0.09)	1.04 (0.99, 1.09)	0.04 (-0.01, 0.08)
**Season**						
**March, April, May**	Ref	Ref	Ref	Ref	Ref	Ref
**June, July, August**	1.00 (0.95, 1.06)	0.00 (-0.06, 0.06)	1.00 (0.95, 1.06)	0.00 (-0.06, 0.06)	1.00 (0.95, 1.06)	0.00 (-0.06, 0.06)
**September, October, November**	1.00 (0.94, 1.07)	0.00 (-0.06, 0.07)	1.00 (0.93, 1.06)	0.00 (-0.07, 0.06)	1.00 (0.94, 1.07)	0.00 (-0.06, 0.07)
**December, January, February**	0.97 (0.91, 1.03)	-0.03 (-0.09, 0.03)	0.94 (0.89, 1.01)	-0.06 (-0.12, 0.10)	0.97 (0.91, 1.03)	-0.03 (-0.09, 0.03)
**Sample collection**						

**1st trimester**	Ref	Ref	Ref	Ref	Ref	Ref
**2nd trimester**	1.01 (0.96, 1.07)	0.01 (-0.04, 0.07)	**1.07 (1.02, 1.13)**	**0.07 (0.02, 0.12)**	1.01 (0.96, 1.07)	0.01 (-0.04, 0.07)
**3rd trimester**	**1.15 (1.09, 1.22)**	0.14 (0.09, 0.20)	**1.24 (1.18, 1.31)**	**0.22 (0.17, 0.27)**	**1.15 (1.09, 1.22)**	**0.14 (0.09, 0.20)**

†Ratios were calculated as the exponent of the beta coefficient. All mycoestrogen concentrations are specific gravity adjusted and missing values were assigned LOD/√2.The mutually adjusted models were adjusted for all considered predictors (i.e. maternal age, early pregnancy BMI, race/ethnicity, parity, use of social services, marital status, fetal sex, season of urine collection), and gestational weeks at urine collection. Category 'Other Races' includes Asian, Pacific Islander, Mixed Race. Bold text indicates significance at $p<0.05$.

Abbreviations: aZOL: alpha-zearalenol, ZEN: zearalenone, SUM: sum of mycoestrogen analytes.

Supplementary Table 6. Ratio and ß estimates of log-transformed aZOL concentrations (ng/ml) by sociodemographic, lifestyle, and perinatal characteristics of UPSIDE participants (N=252) contributing 3 urine samples. †

	Unadjusted		***Adjusted***	
	Ratio (95% CI)†	*ß (95% CI)*	*Ratio (95% CI)†*	*ß (95% CI)*
**Maternal Age (years)**				
**Under 25**	Ref	Ref	Ref	Ref
**25-29**	0.79 (0.61, 1.03)	-0.24 (-0.50, 0.03)	0.71 (0.54, 0.94)	-0.34 (-0.62, -0.06)
**30-34**	0.79 (0.61, 1.03)	-0.23 (-0.49, 0.03)	0.78 (0.57, 1.05)	-0.25 (-0.56, 0.05)
**Over 35**	0.83 (0.60, 1.14)	-0.19 (-0.51, 0.13)	0.80 (0.57, 1.14)	-0.22 (-0.57, 0.13)
**Early Pregnancy BMI**				
**Normal**	Ref	Ref	Ref	Ref
**Overweight**	1.16 (0.95, 1.46)	0.15 (-0.05, 0.38)	1.16 (0.94, 1.42)	0.15 (-0.06, 0.35)
**Obese**	**1.32 (1.09, 1.60)**	**0.28 (0.09, 0.47)**	**1.25 (1.02, 1.52)**	**0.22 (0.02, 0.42)**
**Ethnicity/Race**				
**Non-Hispanic White**	Ref	Ref	Ref	Ref
**Non-Hispanic Black**	1.15 (0.95, 1.40)	0.14 (-0.05, 0.34)	1.06 (0.82, 1.36)	0.06 (-0.20, 0.31)
**Other races**	0.76 (0.55, 1.06)	-0.27 (-0.59, 0.06)	0.79 (0.55, 1.11)	-0.24 (-0.59, 0.10)
**Hispanic**	1.26 (0.95, 1.68)	0.23 (-0.05, 0.52)	1.23 (0.90, 1.68)	0.21 (-0.11, 0.52)
**Education**				
**Less than high school/high school**	Ref	Ref	Ref	Ref
**Some college/college**	0.90 (0.75, 1.09)	-0.10 (-0.29, 0.09)	1.03 (0.82, 1.28)	0.03 (-0.20, 0.25)
**Post-secondary**	**0.74 (0.60, 0.92)**	**-0.30 (-0.51, -0.08)**	0.87 (0.64, 1.17)	-0.14 (-0.44, 0.16)
**Parity**				
**Nulliparous**	Ref	Ref	Ref	Ref
**Multiparous**	**1.22 (1.03, 1.46)**	**0.20 (0.03, 0.38)**	**1.21 (1.01, 1.45)**	**0.19 (0.01, 0.37)**
**Support**				
**None**	Ref	Ref	Ref	Ref
**Any**	1.11 (0.94, 1.31)	0.10 (-0.06, 0.27)	0.90 (0.97, 1.11)	-0.11 (-0.03, 0.10)
**Marital Status**				
**Married/Living as Married**	Ref	Ref	Ref	Ref
**Single/Divorced/Widowed/Separated**	**1.19 (1.00, 1.42)**	**0.17 (0.00, 0.35)**	1.08 (0.86, 1.36)	0.08 (-0.15, 0.31)
**Fetal Sex**				
**Male**	Ref	Ref	Ref	Ref
**Female**	1.09 (0.92, 1.28)	0.09 (-0.08, 0.25)	1.14 (0.96, 1.34)	0.13 (-0.04, 0.29)
**Season**				
**March, April, May**	Ref	Ref	Ref	Ref
**June, July, August**	1.06 (0.88, 1.28)	0.06 (-0.13, 0.25)	0.90 (0.74, 1.06)	-0.11 (-0.30, 0.06)
**September, October, November**	1.16 (0.92, 1.46)	0.15 (-0.08, 0.38)	1.04 (0.84, 1.27)	0.04 (-0.18, 0.24)
**December, January, February**	0.82 (0.66, 1.01)	-0.20 (-0.41, 0.01)	0.83 (0.68, 1.00)	-0.19 (-0.39, 0.00)
**Sample collection**				

**1st trimester**	Ref	Ref	Ref	Ref
**2nd trimester**	**1.38 (1.17, 1.62)**	**0.32 (0.16, 0.48)**	**1.35 (1.15, 1.58)**	**0.30 (0.14, 0.46)**
**3rd trimester**	**2.44 (2.05, 2.83)**	**0.89 (0.72, 1.04)**	**2.48 (2.10, 2.92)**	**0.91 (0.74, 1.07)**

†Ratios were calculated as the exponent of the beta coefficient of a model where the mycoestrogen concentration was the response variable. All mycoestrogen concentrations are specific gravity adjusted. Bold text indicates significance at $p<0.05$. This analysis includes only participants with aZOL concentrations above LOD.

Abbreviations: aZOL: alpha-zearalenol, ZEN: zearalenone, SUM: sum of mycoestrogen analytes.

Supplementary Table 7. Unadjusted ratio and ß estimates of log-transformed urinary mycoestrogen concentrations (ng/ml) by sociodemographic, lifestyle, and perinatal characteristics of UPSIDE participants (N=317) contributing one or more urine samples. †

	ZEN		***aZOL***		***SUM***	
	Ratio (95% CI)†	*ß (95% CI)*	*Ratio (95% CI)†*	*ß (95% CI)*	*Ratio (95% CI)†*	*ß (95% CI)*
**Maternal Age (years)**						
**Under 25**	Ref	Ref	Ref	Ref	Ref	Ref
**25-29**	1.03 (0.93, 1.15)	0.03 (-0.08, 0.14)	0.99 (0.91, 1.07)	-0.02 (-0.10, 0.07)	1.11 (0.91, 1.35)	0.10 (-0.09, 0.30)
**30-34**	0.97 (0.87, 1.07)	-0.03 (-0.14, 0.07)	0.93 (0.86, 1.01)	-0.07 (-0.15, 0.01)	0.98 (0.81. 1.19)	-0.02 (-0.21, 0.18)
**Over 35**	0.98 (0.86, 1.13)	-0.02 (-0.15, 0.12)	0.94 (0.85, 1.05)	-0.06 (-0.16, 0.05)	1.04 (0.81, 1.34)	0.04 (-0.22, 0.29)
**Early Pregnancy BMI**						
**Normal**	Ref	Ref	Ref	Ref	Ref	Ref
**Overweight**	1.05 (0.88, 1.04)	0.05 (-0.13, 0.04)	1.06 (0.99, 1.14)	0.06 (-0.01, 0.13)	1.09 (0.92, 1.29)	0.09 (-0.08, 0.25)
**Obese**	0.96 (0.96, 1.15)	-0.04 (-0.04, 0.14)	1.05 (0.99, 1.12)	0.05 (-0.01, 0.12)	0.98 (0.83, 1.14)	-0.02 (-0.18, 0.14)
**Ethnicity/Race**						
**Non-Hispanic White**	Ref	Ref	Ref	Ref	Ref	Ref
**Non-Hispanic Black**	1.01 (0.95, 1.07)	0.01 (-0.05, 0.07)	1.05 (0.99, 1.13)	0.05 (-0.01, 0.12)	0.98 (0.84, 1.15)	-0.02 (-0.18, 0.14)
**Other Races**	0.96 (0.88, 1.04)	-0.04 (-0.13, 0.04)	0.92 (0.83, 1.02)	-0.08 (-0.18, 0.02)	0.83 (0.64, 1.06)	-0.19 (-0.44, 0.06)
**Hispanic**	0.99 (0.91, 1.07)	-0.01 (-0.09, 0.07)	1.06 (0.96, 1.16)	0.06 (-0.04, 0.15)	1.03 (0.81, 1.30)	0.03 (-0.21, 0.26)
**Education**						
**Less than high school/high school**	Ref	Ref	Ref	Ref	Ref	Ref
**Some college/college**	1.01 (0.93, 1.10)	0.01 (-0.07, 0.09)	0.97 (0.91, 1.03)	-0.03 (-0.10, 0.03)	1.02 (0.87, 1.19)	0.02 (-0.14, 0.17)
**Post-secondary**	0.95 (0.86, 1.04)	-0.05 (-0.15, 0.04)	**0.92 (0.85, 0.99)**	**-0.09 (-0.16, -0.10)**	0.89 (0.75, 1.07)	-0.11 (-0.29, 0.06)
**Parity**						
**Nulliparous**	Ref	Ref	Ref	Ref	Ref	Ref
**Multiparous**	1.04 (0.97, 1.12)	0.04 (-0.03, 0.12)	1.05 (0.99, 1.11)	0.05 (-0.01, 0.11)	1.14 (0.99, 1.32)	0.14 (-0.01, -0.28)
**Support**						
**None**	Ref	Ref	Ref	Ref	Ref	Ref
**Any**	1.00 (0.96, 1.05)	0.00 (-0.05, 0.05)	1.02 (0.97, 1.09)	0.03 (-0.03, 0.08)	0.99 (0.87, 1.14)	-0.01 (-0.14, 0.13)
**Marital Status**						
**Married/Living as Married**	Ref	Ref	Ref	Ref	Ref	Ref
**Single/Divorced/Widowed/Separated**	1.00 (0.96, 1.05)	0.00 (-0.04, 0.05)	**1.07 (1.01, 1.13)**	**0.07 (0.01, 0.12)**	1.07 (0.95, 1.21)	0.07 (-0.05, 0.19)
**Fetal Sex**						
**Male**	Ref	Ref	Ref	Ref	Ref	Ref
**Female**	1.03 (0.99, 1.08)	0.03 (-0.01, 0.08)	1.01 (0.96, 1.07)	0.01 (-0.04, 0.07)	1.02 (0.89, 1.17)	0.02 (-0.11, 0.16)
**Season**						
**March, April, May**	Ref	Ref	Ref	Ref	Ref	Ref
**June, July, August**	1.03 (0.97, 1.09)	0.03 (-0.03, 0.08)	1.02 (0.95, 1.09)	0.02 (-0.05, 0.09)	1.01 (0.84, 1.21)	0.01 (-0.18, 0.19)
**September, October, November**	1.02 (0.96, 1.09)	0.02 (-0.04, 0.09)	1.01 (0.94, 1.09)	0.01 (-0.07, 0.09)	1.07 (0.86, 1.31)	0.06 (-0.15, 0.27)
**December, January, February**	0.97 (0.92, 1.03)	-0.03 (-0.09, 0.03)	0.95 (0.88, 1.02)	-0.05 (-0.13, 0.02)	0.91 (0.75, 1.11)	-0.09 (-0.29, 0.11)

**Sample collection**						
**1st trimester**	Ref	Ref	Ref	Ref	Ref	Ref
**2nd trimester**	1.01 (0.93, 1.10)	0.01 (-0.07, 0.09)	**1.07 (1.01, 1.14)**	**0.07 (0.01,0.13)**	1.08 (0.92, 1.26)	0.08 (-0.08, 0.23)
**3rd trimester**	**1.22 (1.12, 1.32)**	**0.20 (0.12, 0.28)**	**1.27 (1.19, 1.35)**	**0.24 (0.18, 0.30)**	**1.50 (1.28, 1.76)**	**0.41 (0.25, 0.56)**

[†]Ratios were calculated as the exponent of the beta coefficient. All mycoestrogen concentrations are specific gravity adjusted and missing values were assigned LOD/√2.Bold text indicates significance at $p<0.05$.

Abbreviations: aZOL: alpha-zearalenol, ZEN: zearalenone, SUM: sum of mycoestrogen analytes.

Supplementary Table 8. Adjusted ratio and ß estimates of log-transformed urinary mycoestrogen concentrations (ng/ml) by sociodemographic, lifestyle, and perinatal characteristics of UPSIDE participants (N=317) contributing one or more urine samples.[†]

	ZEN		***aZOL***		***SUM***	
	Ratio (95% CI)†	*ß (95% CI)*	*Ratio (95% CI)†*	*ß (95% CI)*	*Ratio (95% CI)†*	*ß (95% CI)*
**Maternal Age (years)**						
**Under 25**	Ref	Ref	Ref	Ref	Ref	Ref
**25-29**	1.02 (0.90, 1.15)	0.01 (-0.11, 0.14)	0.96 (0.88, 1.05)	-0.04 (-0.13, 0.05)	1.02 (0.90, 1.15)	0.01 (-0.11, 0.14)
**30-34**	0.95 (0.84, 1.09)	-0.05 (-0.18, 0.08)	0.92 (0.84, 1.02)	-0.08 (-0.18, 0.02)	0.95 (0.84, 1.09)	-0.05 (-0.18, 0.08)
**Over 35**	0.98 (0.84, 1.15)	-0.02 (-0.18, 0.14)	0.93 (0.83, 1.05)	-0.07 (-0.19, 0.05)	0.98 (0.84, 1.15)	-0.02 (-0.18, 0.14)
**Early Pregnancy BMI**						
**Normal**	Ref	Ref	Ref	Ref	Ref	Ref
**Overweight**	1.05 (0.96, 1.15)	0.05 (-0.04, 0.14)	1.06 (0.99, 1.14)	0.06 (-0.01, 0.13)	1.05 (0.96, 1.15)	0.05 (-0.04, 0.14)
**Obese**	0.93 (0.85, 1.02)	-0.07 (-0.16, 0.02)	1.01 (0.95, 1.09)	0.01 (-0.05, 0.08)	0.93 (0.85, 1.02)	-0.07 (-0.16, 0.02)
**Ethnicity/Race**						
**Non-Hispanic White**	Ref	Ref	Ref	Ref	Ref	Ref
**Non-Hispanic Black**	0.96 (0.86, 1.08)	-0.04 (-0.15, 0.08)	1.01 (0.93, 1.10)	0.01 (-0.07, 0.10)	0.96 (0.86, 1.08)	-0.04 (-0.15, 0.08)
**Other Races**	0.92 (0.80, 1.06)	-0.08 (-0.22, 0.06)	0.93 (0.83, 1.03)	-0.08 (-0.18, 0.03)	0.92 (0.80, 1.06)	-0.08 (-0.22, 0.06)
**Hispanic**	0.93 (0.81, 1.08)	-0.07 (-0.21, 0.07)	1.00 (0.90, 1.11)	0.00 (-0.10, 0.10)	0.93 (0.81, 1.08)	-0.07 (-0.21, 0.07)
**Education**						
**Less than high school/high school**	Ref	Ref	Ref	Ref	Ref	Ref
**Some college/college**	1.01 (0.91, 1.12)	0.01 (-0.09, 0.11)	1.02 (0.95, 1.10)	0.02 (-0.05, 0.10)	1.01 (0.91, 1.12)	0.01 (-0.09, 0.11)
**Post-secondary**	0.95 (0.83, 1.08)	-0.05 (-0.19, 0.08)	0.99 (0.90, 1.10)	-0.01 (-0.11, 0.09)	0.95 (0.83, 1.08)	-0.05 (-0.19, 0.08)
**Parity**						
**Nulliparous**	Ref	Ref	Ref	Ref	Ref	Ref
**Multiparous**	1.07 (0.98, 1.15)	0.06 (-0.02, 0.14)	**1.06 (1.00, 1.12)**	**0.06 (0.00, 0.12)**	1.07 (0.98, 1.15)	0.06 (-0.02, 0.14)
**Support**						
**None**	Ref	Ref	Ref	Ref	Ref	Ref
**Any**	0.95 (0.87, 1.05)	-0.05 (-0.14, 0.05)	0.96 (0.90, 1.03)	-0.04 (-0.11, 0.03)	0.95 (0.87, 1.05)	-0.05 (-0.14, 0.05)
**Marital Status**						
**Married/Living as Married**	Ref	Ref	Ref	Ref	Ref	Ref
**Single/Divorced/Widowed/Separated**	1.06 (0.96, 1.17)	0.06 (-0.04, 0.16)	1.05 (0.97, 1.13)	0.05 (-0.03, 0.12)	1.06 (0.96, 1.17)	0.06 (-0.04, 0.16)
**Fetal Sex**						
**Male**	Ref	Ref	Ref	Ref	Ref	Ref
**Female**	0.99 (0.92, 1.07)	-0.01 (-0.08, 0.06)	1.02 (0.96, 1.07)	0.02 (-0.04, 0.07)	0.99 (0.92, 1.07)	-0.01 (-0.08, 0.06)
**Season**						
**March, April, May**	Ref	Ref	Ref	Ref	Ref	Ref
**June, July, August**	0.97 (0.88, 1.06)	-0.03 (-0.13, 0.06)	0.98 (0.92, 1.05)	-0.02 (-0.08, 0.05)	0.97 (0.88, 1.06)	-0.03 (-0.13, 0.06)
**September, October, November**	1.02 (0.92, 1.14)	0.02 (-0.08, 0.13)	1.00 (0.92, 1.08)	0.00 (-0.08, 0.07)	1.02 (0.92, 1.14)	0.02 (-0.08, 0.13)
**December, January, February**	0.96 (0.87, 1.06)	-0.05 (-0.14, 0.05)	0.95 (0.88, 1.02)	-0.05 (-0.13, 0.02)	0.96 (0.87, 1.06)	-0.05 (-0.14, 0.05)

**Sample collection**						
**1st trimester**	Ref	Ref	Ref	Ref	Ref	Ref
**2nd trimester**	1.00 (0.92, 1.09)	0.00 (-0.08, 0.09)	**1.07 (1.01, 1.14)**	**0.07 (0.01, 0.13)**	1.00 (0.92, 1.09)	0.00 (-0.08, 0.09)
**3rd trimester**	**1.22 (1.12, 1.33)**	**0.20 (0.11, 0.28)**	**1.27 (1.19, 1.35)**	**0.24 (0.18, 0.31)**	**1.22 (1.12, 1.33)**	**0.20 (0.11, 0.28)**

[†]Ratios are calculated as the exponent of the beta coefficient. All mycoestrogen concentrations are specific gravity adjusted and missing values were assigned LOD/√2.The mutually adjusted models are adjusted for all considered predictors (i.e. maternal age, early pregnancy BMI, race/ethnicity, parity, use of social services, marital status, fetal sex, season of urine collection), and gestational weeks at urine collection. Bold text indicates significance at $p<0.05$.

Abbreviations: aZOL: alpha-zearalenol, ZEN: zearalenone, SUM: sum of mycoestrogen analytes.

Supplementary Table 9. Unadjusted ratio and ß estimates of log-transformed placental mycoestrogen concentrations (ng/g) by sociodemographic, lifestyle, and perinatal characteristics of UPSIDE participants (N=271).[†]

	ZEN		***SUM***	
	Ratio (95% CI)	*ß (95% CI)*	*Ratio (95% CI)*	*ß (95% CI)*
**Maternal Age (years)**				
**Under 25**	Ref	Ref	Ref	Ref
**25-29**	2.09 (1.02, 4.32)	0.74 (0.02, 1.46)	1.10 (0.75, 1.62)	0.09 (-0.29, 0.48)
**30-34**	1.34 (0.67, 2.70)	0.30 (-0.40, 0.99)	1.05 (0.72, 1.54)	0.05 (-0.33, 0.43)
**Over 35**	1.90 (0.76, 4.93)	0.64 (-0.28, 1.59)	1.34 (0.82, 2.21)	0.30 (-0.20, 0.79)
**Early Pregnancy BMI**				
**Normal**	Ref	Ref	Ref	Ref
**Overweight**	1.07 (0.59, 1.94)	0.06 (-0.53, 0.66)	1.25 (0.91, 1.71)	0.22 (-0.10, 0.54)
**Obese**	1.13 (0.63, 2.02)	0.12 (-0.46, 0.71)	1.16 (0.85, 1.58)	0.15 (-0.17, 0.46)
**Ethnicity/Race**				
**Non-Hispanic White**	Ref	Ref	Ref	Ref
**Non-Hispanic Black**	0.66 (0.36, 1.20)	-0.42 (-1.01, 0.18)	0.95 (0.69, 1.30)	-0.06 (-0.38, 0.27)
**Other Races**	0.58 (0.23, 1.43)	-0.54 (-1.45, 0.36)	0.67 (0.41, 1.09)	-0.41 (-0.89, 0.08)
**Hispanic**	0.82 (0.37, 1.88)	-0.20 (-1.00, 0.63)	0.76 (0.49, 1.17)	-0.28 (-0.71, 0.16)
**Education**				
**Less than high school/high school**	Ref	Ref	Ref	Ref
**Some college/college**	1.39 (0.79, 2.44)	0.33 (-0.23, 0.89)	1.14 (0.84, 1.54)	0.13 (-0.17, 0.43)
**Post-secondary**	1.05 (0.55, 1.99)	0.05 (-0.59, 0.69)	0.99 (0.70, 1.40)	-0.01 (-0.36, 0.34)
**Parity**				
**Nulliparous**	Ref	Ref	Ref	Ref
**Multiparous**	1.00 (0.60, 1.68)	0.00 (-0.51, 0.52)	0.93 (0.70, 1.22)	-0.07 (-0.35, 0.20)
**Support**				
**None**	Ref	Ref	Ref	Ref
**Any**	0.91 (0.56, 1.48)	-0.09 (-0.58, 0.39)	1.13 (0.87, 1.47)	0.12 (-0.14, 0.39)
**Marital Status**				
**Married/Living as Married**	Ref	Ref	Ref	Ref
**Single/Divorced/Widowed/Separated**	0.64 (0.38, 1.05)	-0.45 (-0.96, 0.05)	0.90 (0.69, 1.18)	-0.11 (-0.38, 0.17)
**Fetal Sex**				
**Male**	Ref	Ref	Ref	Ref
**Female**	1.06 (0.65, 1.73)	0.06 (-0.42, 0.55)	1.18 (0.91, 1.53)	0.17 (-0.09, 0.43)
**Season**				
**March, April, May**	Ref	Ref	Ref	Ref
**June, July, August**	1.63 (0.77, 3.47)	0.49 (-0.26, 1.25)	1.09 (0.73, 1.62)	0.09 (-0.31, 0.48)
**September, October, November**	1.70 (0.85, 3.44)	0.53 (-0.17, 1.24)	**1.49 (1.03, 2.16)**	**0.40 (0.03, 0.77)**
**December, January, February**	0.69 (0.32, 1.48)	-0.37 (-1.13, 0.39)	0.83 (0.55, 1.25)	-0.18 (-0.59, 0.22)
**Length of gestation**				

**Full term**	Ref	Ref	Ref	Ref
**Preterm**	0.62 (0.13, 2.27)	-0.48 (-2.03, 0.82)	1.09 (0.54, 2.18)	0.09 (-0.61, 0.78)

†Ratios were calculated as the exponent of the beta coefficient. The mutually adjusted models were adjusted for all considered predictors (i.e. maternal age, early pregnancy BMI, race/ethnicity, parity, use of social services, marital status, fetal sex, and season of urine collection. For models with ZEN, the response variable is binary (detect/non-detect), for models with sum of mycoestrogen analytes missing values were assigned LOD/√2.

Abbreviations: ZEN: zearalenone, SUM: sum of mycoestrogen analytes.

Supplementary Table 10. Adjusted ratio and ß estimates of log-transformed placental mycoestrogen concentrations (ng/g) by sociodemographic, lifestyle, and perinatal characteristics of UPSIDE participants (N=271).[†]

	ZEN		***SUM***	
	Ratio (95% CI)	*ß (95% CI)*	*Ratio (95% CI)*	*ß (95% CI)*
**Maternal Age (years)**				
**Under 25**	Ref	Ref	Ref	Ref
**25-29**	1.96 (0.80, 4.86)	0.67 (-0.22, 1.58)	1.08 (0.68, 1.72)	0.08 (-0.38, 0.54)
**30-34**	1.21 (0.47, 3.13)	0.19 (-0.75, 1.14)	1.13 (0.69, 1.84)	0.12 (-0.36, 0.61)
**Over 35**	2.33 (0.74, 7.94)	0.85 (-0.30, 2.03)	1.65 (0.91, 2.97)	0.50 (-0.09, 1.09)
**Early Pregnancy BMI**				
**Normal**	Ref	Ref	Ref	Ref
**Overweight**	1.01 (0.53, 1.93)	0.01 (-0.63, 0.66)	1.20 (0.86, 1.67)	0.18 (-0.15, 0.51)
**Obese**	1.11 (0.56, 2.22)	0.11 (-0.57, 0.80)	1.14 (0.81, 1.61)	0.13 (-0.21, 0.48)
**Ethnicity/Race**				
**Non-Hispanic White**	Ref	Ref	Ref	Ref
**Non-Hispanic Black**	0.65 (0.28, 1.48)	-0.44 (-1.27, 0.39)	0.86 (0.56, 1.32)	-0.15 (-0.58, 0.28)
**Other Races**	0.56 (0.20, 1.57)	0.58 (-1.63, 0.45	0.66 (0.38, 1.13)	-0.42 (-0.96, 0.12)
**Hispanic**	0.78 (0.29, 2.15)	-0.25 (-1.25, 0.77)	0.73 (0.44, 1.13)	-0.31 (-0.82, 0.20)
**Education**				
**Less than high school/high school**	Ref	Ref	Ref	Ref
**Some college/college**	0.90 (0.43, 1.89)	-0.10 (-0.85, 0.64)	1.05 (0.72, 1.54)	0.05 (-0.33, 0.43)
**Post-secondary**	0.64 (0.24, 1.70)	-0.44 (-1.44, 0.53)	0.95 (0.58, 1.57)	-0.05 (-0.55, 0.45)
**Parity**				
**Nulliparous**	Ref	Ref	Ref	Ref
**Multiparous**	0.91 (0.51, 1.62)	-0.09 (-0.68, 0.48)	0.85 (0.63, 1.14)	-0.17 (-0.46, 0.13)
**Support**				
**None**	Ref	Ref	Ref	Ref
**Any**	1.18 (0.59, 2.39)	0.16 (-0.53, 0.87)	1.30 (0.91, 1.85)	0.26 (-0.09, 0.62)
**Marital Status**				
**Married/Living as Married**	Ref	Ref	Ref	Ref
**Single/Divorced/Widowed/Separated**	0.60 (0.28, 1.26)	-0.51 (-1.26, 0.23)	0.88 (0.60, 1.28)	-0.13 (-0.51, 0.25)
**Fetal Sex**				
**Male**	Ref	Ref	Ref	Ref
**Female**	0.99 (0.58, 1.68)	-0.01 (-0.54, 0.52)	1.14 (0.87, 1.49)	0.13 (-0.14, 0.40)
**Season**				
**March, April, May**	Ref	Ref	Ref	Ref
**June, July, August**	1.94 (0.86, 4.41)	0.66 (-0.15, 1.48)	1.18 (0.77, 1,79)	0.16 (-0.26, 0.58)
**September, October, November**	1.81 (0.85, 3.87)	0.59 (-0.16, 1.35)	1.46 (0.99, 2.15)	0.38 (-0.01, 0.77)
**December, January, February**	0.66 (0.29, 1.49)	-0.41 (-1.23, 0.40)	0.83 (0.54, 1.28)	-0.19 (-0.61, 0.24)
**Length of gestation**				

**Full term**	Ref	Ref	Ref	Ref
**Preterm**	1.40 (0.30, 7.63)	0.33 (-1.22, 2.03)	1.14 (0.52, 2.51)	0.13 (-0.66, 0.92)

[†]Ratios were calculated as the exponent of the beta coefficient. The mutually adjusted models were adjusted for all considered predictors (i.e. maternal age, early pregnancy BMI, race/ethnicity, parity, use of social services, marital status, fetal sex, and season of urine collection. For models with ZEN, the response variable is binary (detect/non-detect), for models with sum of mycoestrogen analytes missing values were assigned LOD/√2.

Abbreviations: ZEN: zearalenone, SUM: sum of mycoestrogen analytes.

Supplementary Table 11. Adjusted ratio and ß estimates of log-transformed placental mycoestrogen concentrations (ng/g) by sociodemographic, lifestyle, and perinatal characteristics of UPSIDE participants (N=274).[†]

	ZEN		***SUM***	
	Ratio (95% CI)	*ß (95% CI)*	*Ratio (95% CI)*	*ß (95% CI)*
**Maternal Age (years)**				
**Under 25**	Ref	Ref	Ref	Ref
**25-29**	2.12 (0.87, 5.22)	0.75 (-0.14, 1.65)	0.93 (0.59, 1.48)	-0.07 (-0.54, 0.39)
**30-34**	1.30 (0.51, 3.35)	0.26 (-0.67, 1.21)	1.01 (0.62, 1.65)	0.01 (-0.48, 0.50)
**Over 35**	2.56 (0.82, 8.32)	0.94 (-0.20, 2.12)	1.39 (0.77, 2.50)	0.33 (-0.26, 0.92)
**Early Pregnancy BMI**				
**Normal**	Ref	Ref	Ref	Ref
**Overweight**	1.01 (0.58, 2.25)	0.01 (-0.63, 0.65)	1.14 (0.82, 1.58)	0.13 (-0.20, 0.46)
**Obese**	1.13 (0.58, 2.25)	0.12 (-0.55, 0.81)	1.31 (0.92, 1.84)	0.27 (-0.08, 0.61)
**Ethnicity/Race**				
**Non-Hispanic White**	Ref	Ref	Ref	Ref
**Non-Hispanic Black**	0.61 (0.26, 1.39)	-0.50 (-1.33, 0.33)	0.84 (0.55, 1.28)	-0.18 (-0.61, 0.25)
**Other Races**	0.55 (0.19, 1.53)	-0.60 (-1.65, 0.43)	0.60 (0.35, 1.03)	-0.52 (-1.06, 0.03)
**Hispanic**	0.76 (0.28, 2.07)	-0.28 (-1.28, 0.73)	0.92 (0.55, 1.55)	-0.08 (-0.59, 0.44)
**Education**				
**Less than high school/high school**	Ref	Ref	Ref	Ref
**Some college/college**	0.85 (0.40, 1.77)	-0.16 (-0.91, 0.57)	1.16 (0.79, 1.69)	0.15 (-0.23, 0.52)
**Post-secondary**	0.60 (0.23, 1.59)	-0.50 (-1.49, 0.47)	0.98 (0.60, 1.62)	-0.02 (-0.51, 0.48)
**Parity**				
**Nulliparous**	Ref	Ref	Ref	Ref
**Multiparous**	0.90 (0.51, 1.60)	-0.10 (-0.68, 0.47)	0.77 (0.57, 1.03)	-0.26 (-0.56, 0.03)
**Support**				
**None**	Ref	Ref	Ref	Ref
**Any**	0.12 (-0.58, 0.82)	1.13 (0.56, 2.27)	1.18 (0.83, 1.68)	0.16 (-0.19, 0.52)
**Marital Status**				
**Married/Living as Married**	Ref	Ref	Ref	Ref
**Single/Divorced/Widowed/Separated**	-0.48 (-1.22, 0.26)	0.62 (0.30, 1.29)	0.99 (0.68, 1.45)	-0.01 (-0.38, 0.37)
**Fetal Sex**				
**Male**	Ref	Ref	Ref	Ref
**Female**	-0.04 (-0.57, 0.48)	0.96 (0.57, 1.62)	1.17 (0.89, 1.53)	0.16 (-0.11, 0.43)
**Season**				
**March, April, May**	Ref	Ref	Ref	Ref
**June, July, August**	1.90 (0.85, 4.31)	0.64 (-0.16, 1.46)	1.44 (0.95, 2.19)	0.37 (-0.05, 0.79)
**September, October, November**	1.82 (0.85, 3.89)	0.60 (-0.16, 1.36)	**2.73 (1.85, 4.04)**	**1.01 (0.61, 1.40)**
**December, January, February**	0.69 (0.30, 1.55)	-0.37 (-1.19, 0.44)	1.48 (0.96, 2.27)	0.39 (-0.04, 0.82)
**Length of gestation**				

**Full term**	Ref	Ref	Ref	Ref
**Preterm**	1.02 (0.24, 4.62)	0.02 (-1.45, 1.53)	1.08 (0.51, 2.29)	0.08 (-0.67, 0.83)

[†]Ratios were calculated as the exponent of the beta coefficient. The mutually adjusted models were adjusted for all considered predictors (i.e. maternal age, early pregnancy BMI, race/ethnicity, parity, use of social services, marital status, fetal sex, and season of urine collection. For models with ZEN, the response variable is binary (detect/non-detect), for models with sum of mycoestrogen analytes missing values were assigned LOD/√2.

Abbreviations: ZEN: zearalenone, SUM: sum of mycoestrogen analytes.

Supplementary Table 12. Pearson correlations between dietary parameter in the UPSIDE cohort (n=172)

	Energy Intake	UPF%	HEI-2015	Total Fruit	Whole Fruit	Total Veg.	Greens and Beans	Whole Grains	Dairy	Total Proteins	Seafood and Plant Protein	Fatty Acids	Refined Grains	Sodium	Added Sugars	Saturated Fats	% Animal Protein	% Vegetable Protein
**Energy Intake**		0.12	-0.09	-0.11	-0.14	**-0.21**	-0.13	0.00	-0.01	-0.06	-0.01	0.02	0.15	0.06	**-0.26**	-0.07	0.02	-0.02
**UPF%**	0.12		**-0.37**	**-0.33**	**-0.34**	**-0.32**	**-0.30**	0.06	-0.09	**-0.34**	**-0.23**	0.01	**-0.29**	-0.15	**-0.50**	0.10	-0.15	0.15
**HEI-2015**	-0.09	**-0.37**		**0.58**	**0.64**	**0.49**	**0.59**	**0.61**	**0.19**	**0.31**	**0.64**	**0.50**	**0.52**	**0.40**	**0.41**	**0.36**	**-0.39**	**0.39**
**Total Fruit**	-0.11	**-0.32**	**0.58**		**0.88**	**0.21**	**0.18**	**0.18**	0.00	0.08	**0.26**	0.13	**0.24**	**0.29**	**0.22**	**0.33**	**-0.25**	**0.25**
**Whole Fruit**	-0.14	**-0.34**	**0.64**	**0.88**		**0.31**	**0.27**	**0.22**	0.13	0.06	**0.32**	0.12	**0.25**	**0.31**	**0.29**	**0.21**	**-0.32**	**0.32**
**Total Veg.**	**-0.21**	**-0.32**	**0.49**	**0.21**	**0.31**	1.00	**0.60**	0.13	0.08	0.09	**0.29**	**0.18**	0.14	0.02	**0.34**	0.13	**-0.25**	**0.25**
**Greens and Beans**	-0.13	**-0.30**	**0.59**	**0.18**	**0.27**	**0.60**	1.00	**0.23**	0.11	**0.28**	**0.50**	**0.18**	**0.24**	0.14	**0.32**	0.02	**-0.24**	**0.24**
**Whole Grains**	0.00	0.06	**0.61**	**0.18**	**0.22**	0.13	**0.23**	1.00	**0.22**	0.04	**0.32**	**0.18**	**0.30**	**0.19**	**0.18**	**0.18**	**-0.37**	**0.37**
**Dairy**	-0.01	-0.09	**0.19**	0.00	0.13	0.08	0.11	**0.22**	1.00	-0.13	0.06	**-0.34**	0.11	-0.09	**0.23**	**-0.32**	-0.03	0.03
**Total Proteins**	-0.06	**-0.34**	**0.31**	0.08	0.06	0.09	**0.28**	0.04	-0.13	1.00	**0.38**	**0.22**	**0.21**	-0.10	**0.34**	0.01	**0.24**	**-0.24**
**Seafood and Plant Protein**	-0.01	**-0.23**	**0.64**	**0.26**	**0.32**	**0.29**	**0.50**	**0.32**	0.06	**0.38**	1.00	**0.31**	**0.24**	0.15	**0.30**	0.10	**-0.42**	**0.42**
**Fatty Acids**	0.02	0.01	**0.50**	0.13	0.12	**0.18**	**0.18**	**0.18**	**-0.34**	**0.22**	**0.31**	1.00	0.11	0.07	0.08	**0.64**	**-0.31**	**0.31**
**Refined Grains**	0.15	**-0.29**	**0.52**	**0.24**	**0.25**	0.14	**0.24**	**0.30**	0.11	**0.21**	**0.24**	0.11	1.00	**0.38**	-0.03	-0.12	**0.16**	**-0.16**
**Sodium**	0.06	-0.15	**0.40**	**0.29**	**0.31**	0.02	0.14	**0.19**	-0.09	-0.10	0.15	0.07	**0.38**	1.00	**-0.21**	0.09	-0.11	0.11
**Added Sugars**	**-0.26**	**-0.50**	**0.41**	**0.22**	**0.29**	**0.34**	**0.32**	**0.18**	**0.23**	**0.34**	**0.30**	0.08	-0.03	**-0.21**	1.00	**-0.19**	-0.06	0.06
**Saturated Fats**	-0.07	0.10	**0.36**	**0.33**	**0.21**	0.13	0.02	**0.18**	**-0.32**	0.01	0.10	**0.64**	-0.12	0.09	**-0.19**	1.00	**-0.36**	**0.36**
**% Animal Protein**	0.02	-0.15	**-0.39**	**-0.25**	**-0.32**	**-0.25**	**-0.24**	**-0.37**	-0.03	**0.24**	**-0.42**	**-0.31**	**0.16**	-0.11	-0.06	**-0.36**	1.00	**-1.00**
**% Vegetable Protein**	-0.02	0.15	**0.39**	**0.25**	**0.32**	**0.25**	**0.24**	**0.37**	0.03	**-0.24**	**0.42**	**0.31**	**-0.16**	0.11	0.06	**0.36**	**-1.00**	1.00

†The Healthy Eating Index moderation (refined grains. sodium, added sugars, and saturated fats) are such that lower consumption equals a higher score. Dietary measures were averages of two dietary recalls connected in the second trimester. Bold text indicates significance at p<0.05.

Supplementary Table 13. Percent difference (unadjusted) in urinary mycoestrogen concentrations (log-ng/ml) in relation to dietary parameters in the UPSIDE (N=172)[†]

			Percent difference (95% CI)		
**Variable**	**Mean (SD)**	**Range**	**ZEN**	**aZOL**	**SUM**
HEI Total Score	54.9 (13.8)	27.0-92.3	**-1.61 (-2.70, -0.51)**	**-1.79 (-2.97, -0.59)**	**-2.51 (-3.71, -1.29)**
HEI sub-scores - Adequacy					
Total fruit	2.5 (1.6)	0.0-5.0	-4.78 (-13.71, 5.07)	-8.12 (-17.43, 2.24)	-8.56 (-18.23, 2.25)
Whole fruit	2.5 (1.6)	0.0-5.0	-3.74 (-12.65, 6.08)	-6.00 (-15.42, 4.47)	-7.70 (-17.33, 3.06)
Total vegetable	3.1 (1.3)	0.1-5	**-12.23 (-21.99, -1.26)**	**-17.30 (-27.16, -6.11)**	**-18.05 (-28.24, -6.41)**
Greens and beans	2.4 (1.8)	0.0-5.0	-7.38 (-15.09, 1.03)	**-9.57 (-17.71, -0.63)**	**-11.74 (-19.99, -2.64)**
Whole grains	4.2 (3.1)	0.0-10.0	-3.58 (-8.23, 1.32)	-3.70 (-8.76, 1.64)	**-6.56 (-11.63, -1.20)**
Dairy	6.1 (2.7)	0.4–10.0	-2.61 (-8.11, 3.21)	-3.36 (-9.28, 2.95)	-3.93 (-10.07, 2.64)
Total protein	4.2 (1.1)	0.0 -5.0	**-22.30 (-32.67, -10.33)**	**-24.26 (-35.20, -11.46)**	**-26.66 (-37.56, -13.72)**
Seafood and plant protein	2.4 (2.0)	0.0-5.0	-7.32 (-14.34, 0.27)	-8.09 (-15.64, 0.14)	**-9.51 (-17.25, -1.04)**
Fatty acids	4.3 (2.8)	0.0-10.0	-2.34 (-7.58, 3.21)	-0.16 (-6.01, 6.04)	-4.80 (-10.58, 1.35)
HEI sub-scores - Moderation					
Refined grains	5.7 (2.9)	0.0-10.0	4.42 (-0.82, 9.39)	**5.90 (0.28, 11.20)**	5.85 (-0.04, 11.40)
Sodium	5.1 (2.6)	0.0-10.0	-1.00 (-7.20, 4.84)	-2.72 (-9.60, 3.73)	-0.97 (-8.07, 5.66)
Added sugars	7.2 (2.5)	0.0-10.0	**11.30 (5.86, 16.43)**	**13.14 (7.36, 18.56)**	**13.94 (7.96, 19.53)**
Saturated fats	5.0 (2.8)	0.0-10.0	-1.12 (-6.82, 4.28)	-2.74 (-9.06, 3.21)	0.83 (-5.57, 6.85)
**Other dietary measures**					
Energy intake (kcal/day)	2161.6 (321.3)	1261.4-3323.6	0.00 (-0.00, 0.00)	0.00 (-0.00, 0.00)	0.00 (-0.00, 0.00)
% protein from vegetable sources	38.4 (14.2)	11.1-86.9	0.18 (-0.93, 1.30)	0.50 (-0.71, 1.73)	-0.21 (-1.47, 1.06)
% protein from animal sources	61.6 (14.2)	13.1-88.9	-0.18 (-1.28, 0.94)	-0.50 (-1.70, 0.72)	0.21 (-1.05, 1.49)
% kcal from UPF	53.8 (17)	12.3-95.7	**1.25 (0.34, 2.17)**	**1.62 (0.64, 2.61)**	**1.66 (0.63, 2.70)**

[†]The Healthy Eating Index moderation (refined grains. sodium, added sugars, and saturated fats) sub-scores are multiplied by -1 to that higher scores means higher consumption. Dietary measures were averages of two dietary recalls connected in the second trimester. Bold text indicates significance at p<0.05.

Abbreviations: aZOL: alpha-zearalenol, HEI: Healthy Eating Index 2015, kcal: kilocalories, ZEN: zearalenone, SUM: sum of mycoestrogen analytes UPF: ultraprocessed food.

Supplementary Table 14. Percent difference (adjusted) in urinary mycoestrogen concentrations (log-ng/ml) in relation to dietary parameters in the UPSIDE (N=172)[†]

	Percent difference (95% CI)		
**Variable**	**ZEN**	**aZOL**	**SUM**
HEI Total Score	**-2.03 (-3.23, -0.81)**	**-1.84 (-3.15, -0.52)**	-2.77 (-4.08, -1.43)
HEI sub-scores - Adequacy			
Total fruit	-3.34 (-13.08, 7.50)	-6.37 (-16.44, 4.91)	-5.95 (-16.48, 5.90)
Whole fruit	-3.23 (-13.18, 7.87)	-3.95 (-14.51, 7.91)	-5.55 (-16.34, 6.63)
Total vegetable	**-16.42 (-27.15, -4.12)**	**-21.22 (-31.91, -8.86)**	**-21.90 (-32.92, -9.06)**
Greens and beans	-10.03 (-18.18, -1.07)	**-11.17 (-19.76, -1.65)**	**-14.29 (-22.84, -4.78)**
Whole grains	-4.16 (-9.20, 1.16)	-2.55 (-8.07, 3.30)	**-6.14 (-11.62, -0.33)**
Dairy	-2.59 (-8.53, 3.74)	-0.95 (-7.43, 5.98)	-2.61 (-9.23, 4.50)
Total protein	**-24.39 (-34.89, -12.20)**	**-25.94 (-36.92, -13.05)**	**-28.87 (-39.76, -16.02)**
Seafood and plant protein	-7.40 (-14.89, 0.75)	-7.12 (-15.18, 1.70)	**-8.78 (-16.99, 0.24)**
Fatty acids	-4.10 (-9.38, 1.49)	-2.19 (-7.99, 3.98)	**-6.71 (-12.40, -0.65)**
HEI sub-scores - Moderation			
Refined grains	**7.08 (1.86, 12.03)**	**7.31 (1.69, 12.60)**	**8.16 (2.37, 13.61)**
Sodium	-1.71 (-8.14, 4.34)	-3.69 (-10.72, 2.90)	-1.97 (-9.22, 4.79)
Added sugars	**11.20 (5.17, 16.85)**	**12.12 (5.70, 18.10)**	**12.90 (6.27, 19.06)**
Saturated fats	-1.00 (-6.87, 4.55)	-1.90 (-8.27, 4.09)	1.08 (-5.39, 7.14)
Other dietary measures			
Energy intake (kcal/day)			
% protein from vegetable sources	0.43 (-0.74, 1.62)	0.80 (-0.48, 2.09)	0.10 (-1.22, 1.43)
% protein from animal sources	-0.43 (-1.59, 0.75)	-0.79 (-2.04, 0.48)	-0.10 (-1.41, 1.23)
% kcal from UPF	**1.26 (0.29, 2.24)**	**1.60 (0.55, 2.66)**	**1.60 (0.51, 2.71)**

[†]To facilitate interpretation, the Healthy Eating Index moderation (refined grains. sodium, added sugars, and saturated fats) sub-scores are multiplied by -1 to that higher scores means higher consumption. The maximum score for total fruit, whole fruit, vegetable, greens and beans, total protein, seafood and plant protein, is 5 and for whole grains. dairy, fatty acids, refined grains, sodium, added sugars, and saturated fats it is 10. Dietary measures are averages of two dietary recalls connected in the second trimester. Models are adjusted for maternal age, race/ethnicity, parity, education, season of urine collection, marital status, use of social services. Bold text indicates significance at p<0.05.

Abbreviations: aZOL: alpha-zearalenol, HEI: Healthy Eating Index 2015, kcal: kilocalories, ZEN: zearalenone, SUM: sum of mycoestrogen analytes, UPF: ultraprocessed food.

Supplementary Table 15. Percent difference (adjusted for total HEI and covariates) in urinary mycoestrogen concentrations (log-ng/ml) in relation to dietary parameters in the UPSIDE (N=172)†

	Percent difference (95% CI)		
	**ZEN**	**aZOL**	**SUM**
HEI sub-scores - Adequacy			
**Total fruit**	5.13 (-5.82, 18.53)	2.02 (-10.42, 15.03)	7.25 (-5.82, 22.14)
**Whole fruit**	7.25 (-4.88, 20.92)	5.13 (-7.69, 20.92)	9.42 (-3.92, 25.86)
**Total vegetable**	-9.52 (-22.12, 5.13)	**-18.13 (-30.23, -4.88)**	**-13.93 (-26.66, 1.01)**
**Greens and beans**	-3.92 (-9.52, 3.05)	-7.69 (-17.30, 4.08)	-6.76 (-17.30, 4.08)
**Whole grains**	-1.00 (-6.76, 5.13)	1.01 (-4.88, 7.25)	-1.98 (-8.61, 4.08)
**Dairy**	-3.92 (-9.52, 3.05)	-1.98 (-8.61, 4.08)	-3.92 (-10.42, 3.05)
**Total protein**	**-21.34 (-32.97, -7.69)**	**-22.12 (-34.30, -7.69)**	**-24.42 (-36.24, -10.42)**
**Seafood and plant protein**	-2.96 (-11.31, 7.25)	-1.98 (-11.31, 9.42)	-1.00 (-1.98, 9.42)
**Fatty acids**	-1.00 (-6.76, 5.13)	1.01 (-4.88, 7.25)	-1.98 (-8.61, 4.08)
HEI sub-scores – Moderation			
**Refined grains**	**5.13 (0.00, 11.63)**	**6.18 (0.00, 12.75)**	**6.18 (-1.00, 12.75)**
**Sodium**	-2.96 (-8.61, 3.05)	-4.88 (-11.31, 1.01)	-3.92 (-10.42, 2.02)
**Added sugars**	**11.63 (4.08, 18.53)**	**12.75 (5.13, 22.14)**	**12.75 (4.08, 20.92)**
**Saturated fats**	-3.92 (-8.61, 2.02)	-4.88 (-10.42, 1.01)	-2.96 (-8.61, 3.05)

†To facilitate interpretation, the Healthy Eating Index moderation (refined grains. sodium, added sugars, and saturated fats) sub-scores are multiplied by -1 to that higher scores means higher consumption. The maximum score for total fruit, whole fruit, vegetable, greens and beans, total protein, seafood and plant protein, is 5 and for whole grains. dairy, fatty acids, refined grains, sodium, added sugars, and saturated fats it is 10. Dietary measures are averages of two dietary recalls connected in the second trimester. Models are adjusted for maternal age, race/ethnicity, parity, education, season of urine collection, marital status, use of social services, and total HEI score minus the sub-score predictor. Bold text indicates significance at $p<0.05$.

Abbreviations: aZOL: alpha-zearalenol, HEI: Healthy Eating Index 2015, kcal: kilocalories, ZEN: zearalenone, SUM: sum of mycoestrogen analytes.

Supplementary Table 16. Differences in diet quality by BMI and Parity†

Parity	Nulliparous	Multiparous	p-value
n	65	107	
UPF%	56.3	52.3	0.128
HEI-2015	56.7	53.8	0.185
**BMI**	**Low BMI**	**High BMI**	**p-value**
n	79	93	
UPF%	51.1	56.2	0.055
HEI-2015	58.1	52.1	0.005

†Low BMI is <25 kg/m^2 and high BMI>=25 kg/m^2.

Abbreviations: aZOL: alpha-zearalenol, BMI: body mass index, HEI: Healthy Eating Index 2015, kcal: kilocalories, ZEN: zearalenone, SUM: sum of mycoestrogen analytes, UPF: ultraprocessed food.

Supplementary Table 17. Summary of selected recent publications on human biomonitoring of mycoestrogens (years 2014-2022).

Author (Year)	N, Pop.	Location	Matrix, timing	Sample Prep	Separation	Quantitation	LOD (% > LOD)	Concentrations (ng/ml)	Adjusted - Method	Significant Findings
Solfrizzo, M. (2014)	52, children, adults	Italy	Urine, FMV	• Deconjugate: Enzymatic<br>• Purified on a Myco6in1® and OASIS® HLB columns in tandem<br>• No internal standard<br>• Matrix matched calibration curve	UPLC BEH phenyl column (2.1 mm × 150 mm, 1.7 µm particles; Waters)	Triple quadrupole API 5000 system	LOQ:<br>aZOL: 0.03 (100%)<br>bZOL: 0.054 (98%)<br>ZEN: 0.007 (100%)	Median:<br>aZOL: 0.074<br>bZOL: 0.088<br>ZEN: 0.056	No adjustment.	• Prevalent exposure to multiple mycotoxins and ZEN, largely below TDI.
Wallin (2015)	252, adults	Sweden	Spot urine	• Deconjugate: Enzymatic<br>• Purified on a Myco6in1® and OASIS® HLB columns in tandem<br>• No internal standard<br>• Matrix matched calibration curve	UPLC BEH phenyl column (2.1 mm × 150 mm, 1.7 µm particles; Waters)	Triple quadrupole API 5000 system	aZOL: 0.03 (21%)<br>bZO: 0.054 (18%)<br>ZEN: 0.007 (37%)	Mean:<br>aZOL: 0.03<br>bZOL: 0.02<br>ZEN: 0.03	Creatinine<br>aZOL: 0.10<br>bZOL: 0.13<br>ZEN: 0.14	• Prevalent exposure to multiple mycotoxins (69%).<br>• Creatinine adjusted levels higher in women than men.<br>• ZEN, aZOL, bZOL, higher in women than men.
Fleck, S. (2016)	30, pregnant adult females	United States	Serum. Urine, FMW, 24 hour	• Deconjugate: Enzymatic<br>• Isolute SLE+ and supported liquid extraction in 96 well plate<br>• Supelco Titan C18 (100 2.1 mm, 1.9 mm) with a Supelco Titan C18 UPLC guard cartridge<br>• Internal Standard: Isotope	Waters Acquity BEH C18 column (100 2.1 mm, 1.7 mm)	Waters Acquity UPLC System	Not provided, ~50% for ZEN and aZOL, bZAL, bZOL, ZAN were not detected	Mean:<br>ZEN: 0.10<br>aZOL: 0.11	Not applicable.	• Urine to serum ratio of 0.3<br>• No detectable pattern of exposure and food consumption
Ali, N. (2018)	60, adults	Germany	Urine, FMV	• Deconjugate: Enzymatic<br>• Extraction: Immunoaffinity column<br>• Internal Standard: Isotope	Nucleosil® 100-5 C18 HD 125 × 3 mm column	Q-Trap 5500 (ABSciex)	0.10 ng/ml, [LOQ 0.025 ng/ml] (100%)	Median:<br>aZOL: 0.13<br>bZOL: 0.03<br>ZEN: 0.07	Creatinine<br>aZOL: 0.17<br>bZOL: 0.05<br>ZEN: 0.09	• 100% detection of all analytes<br>• aZOL>ZEN>bZOL<br>• In participant who gave multiple samples, higher maximal samples, up to 1.6 ng/ml 'total ZEN'<br>• Most people in cohort below the TDI by EFSA
Ali, N. (2019)	62, adults	Bangladesh (rural and urban)	Urine, FMV Urine, FMV	• Deconjugate: Enzymatic<br>• Extraction: Immunoaffinity column<br>• Internal Standard: Isotope	Nucleosil® 100-5 C18 HD 125 × 3 mm column	Q-Trap 5500 (ABSciex)	0.10 ng/ml, [LOQ 0.025 ng/ml] (100%)	Median<br><br>Summer<br>aZOL: 0.194<br>bZOL: 0.013<br>ZEN: 0.021<br>Winter<br>aZOL: 0.183<br>bZOL: 0.011<br>ZEN: 0.027	Creatinine<br><br>Summer<br>aZOL: 0.993<br>bZOL: 0.023<br>ZEN: 0.114<br>Winter<br>aZOL: 0.338<br>bZOL: 0.029<br>ZEN: 0.064	• Higher aZOL than ZEN<br>• Higher levels in the winter<br>• Analyte composition similar between sexes

	20, pregnant female adults							Median:<br>aZOL: 0.052<br>bZOL: 0.032<br>ZEN: 0.156	Creatinine<br>aZOL: 0.516,<br>bZOL: 0.148<br>ZEN: 0.185	• bZOL is more prevalent in pregnant women<br>• bZOL is higher in pregnant than non-pregnant
Mauro, T. (2018)	48, healthy female adults	United States	Serum, fasting	• Free and total<br>• Deconjugate: Enzymatic	Hypersil Gold C18, 50×2.1mm	Thermo LTQ	0.07 ng/ml<br>aZOL: 63%<br>aZAL: 75%<br>bZOL: 40%<br>bZAL: 17%<br>ZAN: 94%<br>ZEN: 100%	Mean – conjugated:<br>aZOL: 0.44<br>aZAL: 0.30<br>bZOL: 0.23<br>bZAL: 0.18<br>ZAN: 0.20<br>ZEN: 0.64		• ZEN was lower in overweight/obese women<br>• Those with highest meat intake had highest concentrations<br>• Dairy and grains not associated with ZEN
Carballo, D. (2021)	40, adults	Spain	Urine, FMV	• Salting-out liquid-liquid extraction	Gemini® NX-C18 (3 μM, 150 × 2 mm ID<br>guard column C18 (4 × 2 mm, ID; 3 μM)	6540 Agilent Ultra-High-Definition Accurate-Mass q-TOF-MS	ZEN: 0.33 ng/ml (40%)<br>aZOL: 0.33 ng/ml (43%)	Mean:<br>ZEN: 6.7 ng/ml<br>aZOL: 27.44 ng/ml		• OTA and ZEN were prevalent in urine
Kyei, N. (2022)	439, pregnant female adults	Banglad esh (rural)	Urine, FMV	• Free<br>• 'Dilute and shoot'<br>• Refer: Gerding et al. (2014)	NUCLEODUR® C_{18} Pyramid column (3 μm, 2.0 × 150 mm, MACHEREY-NAGEL, Düren, Germany) equipped with a C_{18} EC guard column<br>(2 mm x 4 mm, MACHEREY-NAGEL)	AB SCIEX QTRAP®5500 mass spectrometer	ZEN: 0.7 ng/ml<br>ZAN: 1.25 ng/ml<br>aZOL-glu-14: 3<br>bZOL-glu-14: 7<br>(1%)	Only one positive sample	Specific gravity, creatinine (individually)	• This study examined multiple mycotoxins, exposure to OTA and citrinin was high, but ZEN was only found in one sample.
Duarte, S. (2022)	37, lactating adult females	Angola	Breast milk	• Deconjugate: Enzymatic	Competitive ELISA		ZEN: 0.060 ng/ml	Mean:<br>0.436±0.36		• 100% of samples contained ZEN and OTA<br>• Significant association of eating cookies with ZEN
Zhang, S. (2022)	199, children and adults	China	Urine, FMV	• Deconjugate: Enzymatic<br>• Internal Standard: Isotope<br>• Oasis ® PRiME HLB μElution Plate	CORTECS™UPLC® C18 column (2.1 × 100 mm, 1.6 μm)	Xevo® TQ-S tandem quadrupole mass spectrometer	LOD ng/ml<br>aZOL: 0.04 (26%)<br>aZAL: 0.04<br>bZOL: 0.06 (24%)<br>bZAL: 0.02<br>ZAN: 0.03<br>ZEN: 0.02 (88%)	Median:<br>aZOL: 0.02<br>bZOL: 0.03<br>ZEN: 0.218		• aZOL were found to be lower than bZOL<br>• First study examining relationship between exposure predicted by diet and urinary levels

Abbreviations: aZOL: alpha – zearalenol, aZOL-glu-14: alpha-Zearalenol-14-glucuronide, bZOL: beta-zearalenol, bZOL-glu-14: beta-Zearalenol-14-glucuronide, EFSA: European Food Safety Authority, FMV: first morning void, TDI: tolerable daily intake, OTA: ochratoxin
